# The 5′ Cap Epitranscriptome and Beyond: Natural and Engineered 5′ Cap Modifications for Optimizing mRNA Therapeutics and Functional Studies

**DOI:** 10.1002/cmdc.202500826

**Published:** 2026-01-31

**Authors:** Greta Charlotte Dahm, Melissa Pieper, Helena Schepers, Andrea Rentmeister

**Affiliations:** ^1^ Department of Chemistry Ludwig‐Maximilians‐Universität Butenandtstr. 5‐13 81377 München Germany; ^2^ Institute for Biochemistry University of Münster Corrensstr. 36 48149 Münster Germany

**Keywords:** 5′ cap, AdoMet, methylation, mRNA, RNA modifications

## Abstract

Eukaryotic mRNAs made by in vitro transcription have emerged as medical modalities for vaccination and protein replacement therapy. The 5′ cap is an essential feature of eukaryotic mRNAs providing stability, reducing immunogenicity, and serving as starting point for translation initiation. The “cap epitranscriptome” comprises several natural 5′ cap modifications that can impact mRNA interactions and fate. Manipulating this privileged structure provides a powerful handle to optimize mRNA properties and to build a toolbox for investigating and controlling mRNA‐related processes. In this article, the impact of natural 5′ cap modifications on mRNA translation, immunogenicity, and stability is highlighted. Then, it is shown how non‐natural 5′ cap modifications have been used to manipulate and optimize various mRNA properties. Finally, non‐natural modifications can equip mRNA with reactive handles, which provide a toolbox for studying interactions and controlling the function of mRNAs.

## Graphical abstract

5′ Cap modifications can be introduced via enzymes or chemical synthesis. Natural and non‐natural 5′ cap structures affect mRNA properties, including translation (T), stability (S), and/or immunogenicity (I).

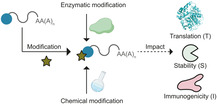

## 1 Introduction

The potential for utilizing eukaryotic mRNA in medical applications has advanced significantly in recent years. Decades of fundamental research in RNA biochemistry laid the groundwork for mRNA as a therapeutic modality. Then, within only one year after the genome sequence of SARS‐CoV‐2 was published, Moderna (mRNA‐1273) and Pfizer/BioNTech (Bnt162b2) obtained emergency use authorization from the U.S. Food and Drug Administration (FDA) for their clinical‐grade Covid‐19 vaccines.^[^ ^1^ ^,^ ^2^ ^]^ In addition to these well‐known mRNA‐based vaccines, mRNA is being tested for protein replacement therapies as well as for personalized medicine in several clinical trials.^[^ ^3^, ^4^, ^5^ ^]^ Historically, for many years, mRNA had not been considered a therapeutic agent. The drawbacks of limited stability in cells and degradation by almost ubiquitous RNases together with the immunogenicity of foreign RNAs applied to cells were considered unsurmountable by many scientists. This has changed by the impactful work of Katalin Karikó and Drew Weissmann on natural modified nucleotides in mRNAs to reduce immunogenicity, awarded with Nobel prize for medicine in 2023 together with improved delivery methods.^[^ ^6^, ^7^, ^8^ ^]^

The 5′ cap is the most important canonical modification and a hallmark of eukaryotic mRNA. It was already discovered in 1975^[^ ^9^, ^10^, ^11^ ^]^ and found to be imperative for translation initiation.^[^ ^12^ ^]^ The recent rise of mRNAs as medical modality has kindled interest in better understanding the 5′ cap epitranscriptome and leveraging it to make synthetic 5′ caps with improved properties for cellular and potentially even medical applications.

In this article, we summarize the biogenesis of mRNA and focus on the role of the 5′ cap for various processes in the cell. We will then explain how mRNAs with a 5′ cap can be made in vitro.

### 1.1 The 5′ Cap Functions in Translation, Immunogenicity, and Stability

**Figure 1:**
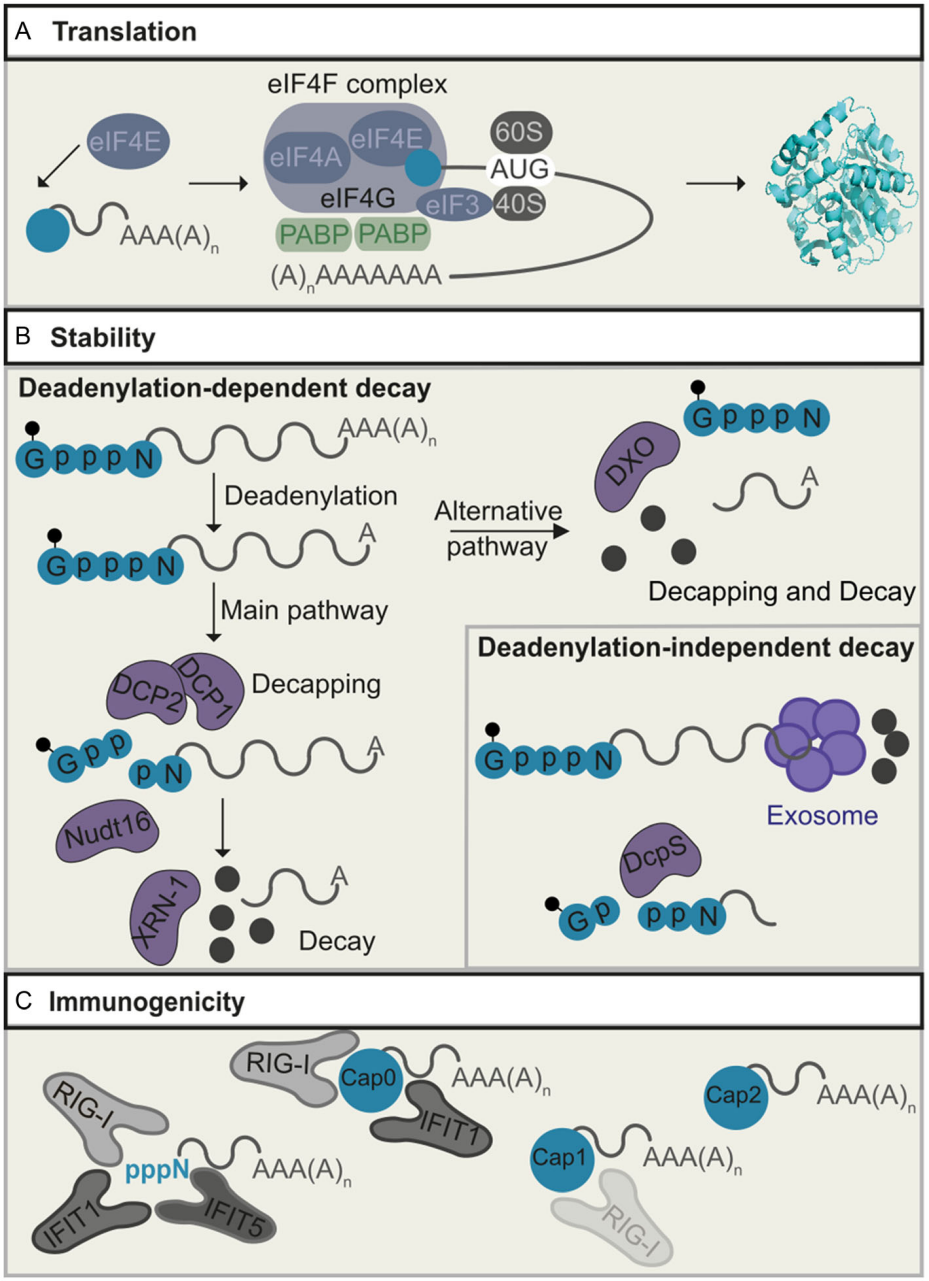
The role of the 5′ cap of mRNA (m^7^GpppN) in translation, stability, and immunogenicity. A) In the cytoplasm, the eukaryotic translation initiation factor 4E (eIF4E) binds to the 5′ cap (blue circle) and recruits other translation initiation factors (eIFs) and poly(A)‐binding proteins PABPs. The recruitment of the ribosomal subunits then initiates translation. B) The 5′ cap (m^7^GpppN: blue circle with small black dot for methylation) stabilizes mRNA by preventing degradation by 5′ exonucleases. Two pathways are involved in the degradation of mRNA after deadenylation. Deadenylation‐dependent decay (from the 5′ end) includes either decapping enzyme 1/2 (Dcp1/2) or Nudt16, followed by exonuclease XRN1 or DXO, which combines decapping and exonucleolytic activity. Deadenylation‐independent decay from the 3′ end by the decapping scavenger enzyme (DcpS). Small black dots indicate methylation in m^7^G, large black dots indicate liberated nucleotides. C) The 5′ cap facilitates discrimination between self and non‐self mRNAs. The receptor families RIG‐1‐like receptors and IFN‐induced proteins with tetratricopeptide repeats (IFIT) are part of the immune system and recognize uncapped and cap 0, cap 1‐mRNAs, whereas cap 2‐mRNAs are not recognized.

The 5′ cap is involved in mRNA processing, export, stability, immunogenicity, and translation.^[^ ^13^, ^14^, ^15^, ^16^, ^17^, ^18^ ^]^ We will focus on the 5′ cap's known effects on translation, immunogenicity, and stability on a molecular basis (**Figure** **1**).

The 5′ cap is the starting point of translation initiation.^[^ ^16^ ^,^ ^19^ ^]^ In the cytoplasm, the eukaryotic translation initiation factor 4E (eIF4E) first interacts with the 5′ cap, before additional translation initiation factors are recruited to form the heterotrimeric complex eIF4F and initiate translation^[^ ^20^ ^,^ ^21^ ^]^ (Figure 1A). Modifications at the 5′ cap can drastically affect binding to eIF4E and consequently the amount of protein produced from an mRNA.^[^ ^22^ ^,^ ^23^ ^]^

The 5′ cap increases the stability of mRNA by preventing degradation by 5′‐exonucleases.^[^ ^15^ ^]^ Eukaryotic mRNAs can vary in their half‐lives from few hours to days,^[^ ^24^, ^25^, ^26^ ^]^ but are generally much more stable than prokaryotic mRNAs that do not possess a 5′ cap and are turned over within minutes.^[^ ^27^, ^28^, ^29^ ^]^ In mammals, the main mRNA decay pathway starts by deadenylation, that is, shortening of the poly(A) tail (Figure 1B). With the loss of the poly(A)‐binding protein (PABP), the 5′ cap becomes accessible to decapping enzymes, such as Dcp1/2 and Nudt16, which hydrolyze the 5′ cap between the α‐ and β‐phosphates and release m^7^GDP, resulting in 5′ monophosphorylated mRNA.^[^ ^30^ ^,^ ^31^ ^]^ The exonuclease XRN1 can then rapidly degrade the monophosphorylated mRNA from the 5′ end^[^ ^32^ ^]^ (Figure 1B). DXO is another decapping enzyme that, unlike Dcp2, cleaves off the entire 5′ cap structure and also has 5′–3′ exonucleolytic activity, through which it can eliminate RNA designated for degradation^[^ ^33^ ^]^ (Figure 1B). It is preferably active on incompletely capped pre‐mRNAs.^[^ ^34^ ^]^ DcpS is the decapping scavenger enzyme, which “decaps” short mRNA after the alternative 3′ to 5′ exoribonucleolytic decay (Figure 1B). It cleaves the cap structure between the β‐ and γ‐position.^[^ ^35^ ^,^ ^36^ ^]^

Finally, the 5′ cap also impacts immunogenicity of an mRNA (Figure 1C). The innate immune system of humans detects pathogenic structures.^[^ ^37^ ^]^ Several receptors and proteins have been described to recognize foreign mRNA, in particular double stranded or uncapped RNAs originating from viruses, like the melanoma differentiation‐associated gene 5 (MDA5), which recognizes long dsRNA with higher‐order structures.^[^ ^38^, ^39^, ^40^ ^]^ Only few receptors and proteins have been confirmed to interact with the 5′ cap. These include retinoic‐acid‐inducible gene 1 (RIG‐1) and interferon‐induced proteins with tetratricopeptide repeats‐1 and ‐5 (IFIT1 and IFIT5)^[^ ^13^ ^,^ ^14^ ^,^ ^41^ ^]^ (Figure 1C).

### 1.2 Biogenesis of 5′ Capped mRNA

**Figure 2:**
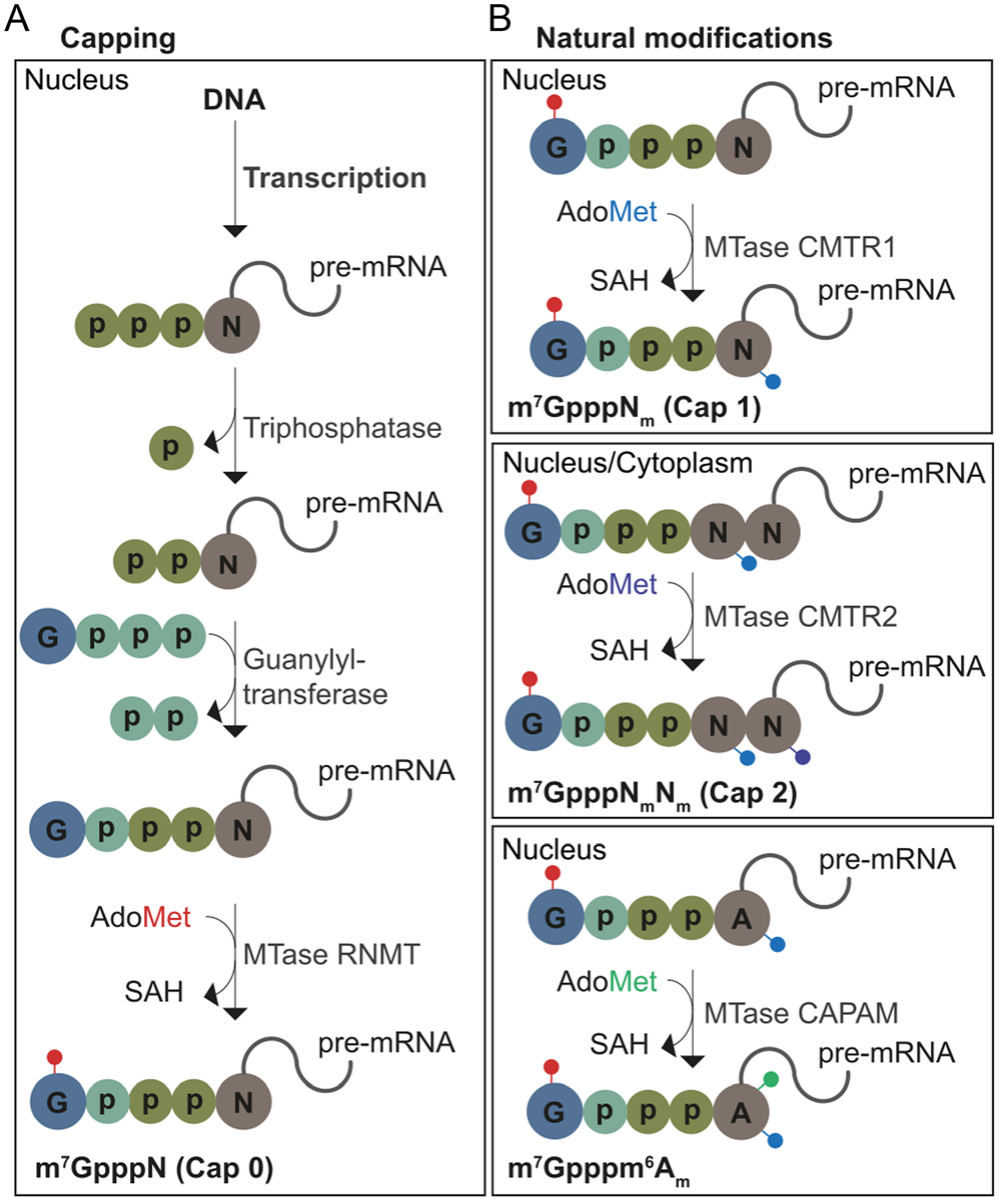
Canonical capping of pre‐mRNA in the nucleus. A) Co‐transcriptional capping is performed in three enzymatic steps. First, the γ‐phosphate is removed from the 5′ end of the TSN by RNA triphosphatase generating 5′ diphosphate RNA. Next, a guanylyltransferase incorporates GMP from GTP to the RNA diphosphate, forming a guanosine cap (GpppN, by which N indicates the TSN). GpppN is further methylated at the *N*7G by mRNA cap guanine‐N7 methyltransferase (RNMT), which results in the cap 0 structure (m^7^GpppN). B) Natural cap modifications comprise additional methylations of the cap structure. The 2′‐*O* position of the first (m^7^GpppN_m_) or additionally the second (m^7^GpppN_m_pN_m_) transcribed nucleotide can be methylated by methyltransferases (MTases) CMTR1 or CMTR2, respectively. CMTR2 can be found both in the nucleus and cytoplasm. Additionally, the *N* ^6^ position of the TSN adenosine can be methylated by the cap‐specific adenosine *N* ^6^‐methyltransferase (CAPAM). Besides capping and cap modifications, pre‐mRNA is further processed by splicing and polyadenylation. Fully processed mature mRNA is exported from the nucleus to the cytoplasm.

The capping of eukaryotic mRNAs occurs cotranscriptionally at the 5′ end of 20–30 nucleotide long nascent RNA polymerase II transcripts.^[^ ^42^ ^,^ ^43^ ^]^ An RNA triphosphatase removes the terminal 5′ γ‐phosphate group from the transcription start nucleotide (TSN), leaving 5′ diphosphate RNA. A guanylyltransferase then catalyzes the transfer of guanosine monophosphate (GMP) from a guanosine triphosphate (GTP) to the RNA diphosphate end. The result is a GpppN‐RNA, that is, a 5′‐5′ linked guanosine at the TSN (N) followed by the RNA.^[^ ^20^ ^]^ The GpppN is further methylated at the *N*7G by mRNA cap guanine‐*N*7 methyltransferase (RNMT), leading to formation of m^7^GpppN, which is cap 0 (**Figure** **2**). RNMT, as well as other methyltransferases, uses *S*‐adenosyl‐L‐methionine (AdoMet/SAM) as cofactor.^[^ ^44^, ^45^, ^46^ ^]^

**Figure 3:**
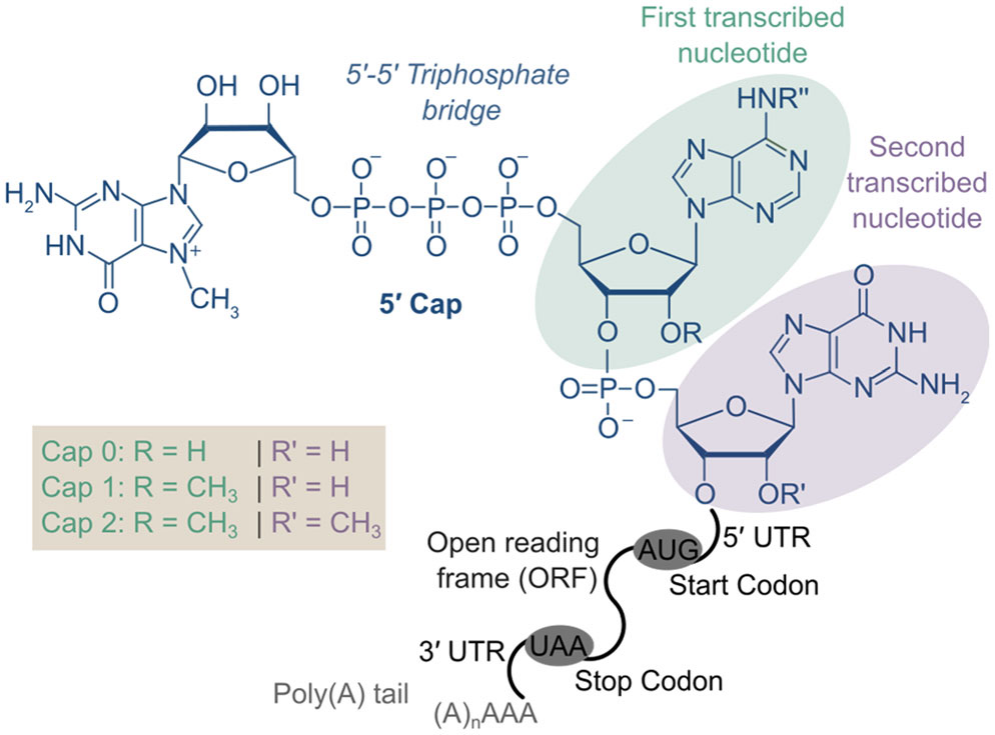
Important elements of fully processed mature eukaryotic mRNA: The 5′ cap (highlighted in blue), a 5′ untranslated region (UTR), the open reading frame (ORF), marked by start and stop codon, the 3′ UTR, and the 3′ poly(A) tail. The chemical structure of the 5′ cap is shown. It consists of a guanosine, which is methylated at the *N* *7* position. This guanine is linked to the TSN (here adenosine, green circle) via a 5′‐5′ triphosphate bridge. Depending on the 2′‐*O*‐methylation status of the ribose of the TSN as well as the second transcribed nucleotide (purple circle) a cap 0, cap 1, or cap 2 structure is formed.

Cap 0 is the predominant 5′ cap structure in yeast. In higher eukaryotes, such as mammals, mRNA is further methylated. The 2′‐*O* position of the TSN is methylated by the cap methyltransferase 1 (CMTR1) to m^7^GpppN_m_, which is cap 1.^[^ ^47^ ^]^ Since hMTr1 (human CMTR1) is also active on GpppG‐mRNA, methylation might be performed even prior to methylation by RNMT.^[^ ^47^ ^]^ Additional methylation at the ribose of the second transcribed nucleotide leads to m^7^GpppN_m_pN_m_, which is cap 2. The reaction is catalyzed by CMTR2, which—in contrary to the other cap methyltransferases—is also found and active in the cytoplasm^[^ ^48^, ^49^, ^50^ ^]^ (**Figure** 2 and **3**). The presence of cap 1 increases the hMTr2 (human CMTR2) activity but is not required.^[^ ^50^ ^]^

It was shown that levels of cap 1‐mRNAs are organ‐ and cell‐specific, indicating a variable regulation of mRNA cap formation.^[^ ^51^, ^52^, ^53^ ^]^ For the production of mRNAs in vitro, it is important to point out that both cap 0 and cap 1 analogs are commercially available and can be co‐transcriptionally incorporated during in vitro transcription (IVT).

An important parameter of the 5′ cap is the TSN. Most mammalian m^7^G‐capped mRNAs start with A_m_, which often is additionally methylated at the *N* ^6^ position to m^6^A_m_ by the cap‐specific adenosine *N* ^6^‐methyltransferase (CAPAM)^[^ ^48^ ^,^ ^51^ ^,^ ^54^ ^]^ (Figure 2). G_m_ or C_m_ can also be found as TSN in mammalian mRNA, while U_m_ as TSN is about 100‐times less prominent.^[^ ^55^ ^]^

After capping including the various 5′ cap modifications (except for cap 2), pre‐mRNA is further processed by splicing and polyadenylation. Fully processed mRNA is then exported to the cytoplasm.

Capping is predominantly performed co‐transcriptionally, however, cytoplasmic capping can occur as a repair mechanism. A guanylyltransferase forms a complex with a 5′‐monophosphate kinase, capping 5′‐monophosphorylated RNAs.^[^ ^56^ ^,^ ^57^ ^]^ Furthermore, Despic and Jaffrey found that through conversion from cap 1 to cap 2 in the cytosol, cap 2 is enriched on long‐lived mRNAs.^[^ ^53^ ^]^ Other natural 5′ caps, like NAD, flavin adenine dinucleotide, UDP‐Glc and UDP‐GlcNAc exist and are currently investigated,^[^ ^51^ ^,^ ^58^ ^,^ ^59^ ^]^ however, they are beyond the scope of this review.

### 1.3 In Vitro Production of 5′ Capped mRNAs

To understand how various 5′ cap modifications affect mRNA properties, the corresponding 5′ capped mRNAs need to be accessible in high purity.

Transcriptional priming with the desired 5′ cap during IVT with phage RNA polymerases (e.g., T7, SP6) allows the production of mRNAs with different 5′ caps. The respective 5′ cap analogs can be chemically synthesized and several ones are commercially available (m^7^GpppG, anti‐reverse cap analog (ARCA), CleanCap). These cap analogs can mimic natural 5′ caps (cap 0, cap 1) or can bear additional modifications at various positions.

Synthetic access to 5′ cap analogs provides a cornerstone for therapeutic mRNAs, including the Covid‐19 vaccine developed by Pfizer/BioNTech.^[^ ^1^ ^,^ ^2^ ^]^ Here, a commercially available cap 1, the trinucleotide m^7^GpppA_m_G (referred to as CleanCap) was incorporated.^[^ ^60^ ^,^ ^61^ ^]^ The co‐transcriptional incorporation of CleanCap results in high yields of cap 1‐mRNAs and high capping efficiency of up to 90%–99%.^[^ ^60^ ^]^ These high capping efficiencies can be explained by the fact that trinucleotide caps allow for possible base pairing of two nucleotides with the DNA template, which is thermodynamically more stable than a single base pair.^[^ ^55^ ^]^

Besides the co‐transcriptional incorporation of CleanCap or other cap analogs, 5′ caps can be added enzymatically to a ppp‐RNA using capping enzymes, such as the vaccina capping enzyme.^[^ ^14^ ^]^ Modification as for instance to cap 1 can also be performed enzymatically,^[^ ^62^ ^]^ as it was the case for the Covid‐19 vaccine developed by Moderna.^[^ ^63^ ^]^ Thus, this post‐transcriptional capping approach using capping enzymes is an alternative route, where uncapped mRNA with a 5′ triphosphate is prepared via IVT and then further modified. For both approaches (chemical synthesis or the use of capping enzymes) additional post‐transcriptional modification of the 5′ cap is possible by using methyltransferases and appropriate cosubstrates (reviewed in more detail in Refs. [64,65]). Taken together these approaches provide access to study mRNAs with naturally occurring and non‐natural 5′ caps.

## 2 Naturally Occurring 5′ Cap Modifications and Their Effect on Stability, Translation, and Immunogenicity

In this chapter, we focus on natural modifications of the 5′ cap and their impact on mRNA stability, translation, and immunogenicity in cells. We will refer to “modifications” of the 5′ cap based on the cap 0 structure (m^7^GpppN).

### 2.1 Stability

Stability is a major limitation for biotechnological and medical application of mRNAs. Hence, increasing the lifetime of mRNAs is of paramount importance. Extensive academic and industrial research has led to codon optimization, increased G/C content, optimization of the poly(A) tail, and the incorporation of modified nucleosides. All of these changes contribute—in addition to other effects—to improved mRNA stability^[^ ^66^, ^67^, ^68^, ^69^, ^70^ ^]^ and have been summarized in excellent reviews.^[^ ^71^, ^72^, ^73^ ^]^

We focus on the effect of modifications of the 5′ cap on mRNA stability. Interestingly, additional 5′ cap methylations have distinct effects on the various decapping enzymes mentioned above.

The 2′‐*O*‐methylation of the TSN (cap 1) increases pre‐mRNA stability by abolishing recognition through the exoribonuclease DXO.^[^ ^74^ ^]^ Recently, additional methylation at the second ribose in cap 2 was reported to have similar effects on DXO activity as cap 1. Here, the presence of the 2′‐*O*‐methyl group at the TSN or at both, the first and second transcribed nucleotides, protects transcripts from hDXO activity compared to cap 0 mRNA.^[^ ^75^ ^]^

Decapping by Dcp2, however, is not affected by the methylation of the TSN or second transcribed nucleotide.^[^ ^55^ ^,^ ^75^ ^]^ The Dcp1/Dcp2 heterodimer (with Dcp2 as catalytically active subunit), is the main factor responsible for cap elimination from transcripts designated for degradation in mammals.^[^ ^76^ ^]^ Susceptibility to hDcp2 activity in vitro is similar for cap 0, cap 1, and cap 2.^[^ ^75^ ^]^

The *N* ^6^‐methylation of adenosine as TSN by CAPAM could also affect mRNA stability. Interestingly, the role of cap‐specific m^6^A_m_ in mRNA stability and translation has not been fully clarified to date and is controversially discussed in literature. First studies observed a stabilizing effect of cap‐specific m^6^A_m_ in comparison to A_m_, using transcriptome‐wide mapping.^[^ ^77^ ^,^ ^78^ ^]^ When mRNAs containing m^7^GpppA_m_ or m^7^Gppp(m^6^A_m_) were prepared in vitro, this effect is partially explained by the resistance to the mRNA decapping enzyme Dcp2.^[^ ^77^ ^]^ In contrast, other studies observed no correlation between mRNA stability and m^6^A_m_. After a knockdown of the RNA demethylase FTO (fat mass and obesity‐associated protein), m^6^A_m_ containing transcripts showed significantly lower transcript level changes compared to other transcripts.^[^ ^79^ ^]^ A stabilizing role of m^6^A_m_ would lead to elevated m^6^A_m_ levels.^[^ ^79^ ^]^ Another study with CAPAM knockout melanoma cells showed that the stability of m^6^A_m_ transcripts is not significantly affected by a loss of the methylation.^[^ ^80^ ^]^

### 2.2 Translation

The identity of the TSN affects the amount of protein produced even without further methylations. Cap 0‐ and cap 1‐RNAs with adenosine (or A_m_) as TSN yield more protein in mice and human cells than the respective RNAs with any of the other three nucleosides, when mRNAs are produced by IVT.^[^ ^55^ ^,^ ^60^ ^]^ Therefore, varying the TSN can be useful for finetuning the protein output.

The methylation status at various positions of the 5′ cap strongly affects translation. Most importantly, the methylation of the *N*7 position of the 5′ cap guanosine increases the binding affinity to the translation initiation factor eIF4E by ≈100‐fold.^[^ ^81^ ^,^ ^82^ ^]^ This methylation is thus a prerequisite for translation initiation.

The effect of additional methylations, that is, cap 1 and cap 2, is more subtle and often cell‐dependent. Cap 1‐mRNAs (made by IVT with the respective trinucleotide cap) increased translational output compared to cap 0‐mRNAs. Interestingly, the effect was only observed in dendritic cells (JAWS II), but not in HeLa or 3T3‐L1 cells.^[^ ^55^ ^]^ Binding studies revealed similar affinity of eIF4E for cap 0 and cap 1 as trinucleotides (around 25–50 µM).^[^ ^55^ ^]^ Additional methylation of only the second transcribed nucleotide (m^7^GpppApG_m_) led to a twofold higher protein production in A549 cells, whereas, the translational output was strongly decreased in JAWS II and THP‐1 cells. Finally, the translation of cap 2‐mRNAs (m^7^GpppA_m_G_m_) was not increased in A549 cells, but at the level of cap 0‐mRNA.^[^ ^75^ ^]^ Taken together, the methylation status of the 5′ cap is clearly important for the translational output, however, the effect depends on the cell line used. More comprehensive studies with identical constructs but different 5′ cap modifications are necessary to understand the phenomenon. It is also plausible that the observable “amount of protein produced” comprises a blackbox of various parameters. To fully understand the effect of the 5′ cap methylation status, it would therefore be helpful to dissect underlying processes, such as ribosome load, stability, and activation of various pathways in different cell lines.

Additional methylation at the *N* ^6^ position of TSN (m^6^A/m^6^A_m_) also affects protein output, but there is controversy in the literature not only about mRNA stability data regarding these modifications but also about mRNA translation.^[^ ^54^ ^,^ ^78^ ^,^ ^80^ ^]^ On the one hand, mRNA containing m^6^A_m_ has similar binding affinities for eIF4E as mRNA with the TSN A_m_.^[^ ^54^ ^]^ Ribosome profiling experiments in CAPAM knockout cells found no significant difference on ribosome distribution on mRNAs, however, Akichika et al. state that m^6^A_m_ has the ability to up‐regulate cap‐dependent translation.^[^ ^54^ ^]^ One reason for that might be that cap‐binding proteins other than eIF4E are involved in this process.^[^ ^54^ ^]^

Other groups prepared mRNAs via IVT and did not see a strong increase in translation after transfection of cells.^[^ ^55^ ^,^ ^75^ ^,^ ^80^ ^,^ ^83^ ^]^ Sikorski et al. observed slightly higher protein production for cap 1‐mRNA containing m^6^A_m_ as TSN in HeLa and JAWS II cells, whereas this effect was not observed for cap0‐mRNA containing m^6^A as TSN.^[^ ^55^ ^]^ In addition, the incorporation of the TSN m^6^A in cap 0‐mRNAs decreased the translational output in comparison to adenosine, both in murine JAWS II and 3T3‐L1 cells, whereas, the translational output of this m^7^Gppp(m^6^A)‐mRNA in human THP‐1 and A549 cells was not affected by this modification.^[^ ^75^ ^]^ The decreased translational output might be explained by lower binding affinities for eIF4E and m^7^Gppp(m^6^A)‐mRNAs in contrast to their unmethylated counterparts.^[^ ^75^ ^]^ This study shows that the effects of the methylation status of cap 0‐, cap 1‐, and cap 2‐mRNAs are markedly cell line dependent.^[^ ^75^ ^]^ In human HEK‐NF‐κB and HeLa cells, it was found that m^7^Gppp(m^6^A_m_)‐mRNA decreased the translational output compared to mRNAs unmethylated at the *N* ^6^ position of TSN.^[^ ^83^ ^]^ Also, in melanoma cells, the translation of eGFP‐ and luciferase‐m^7^Gppp(m^6^A_m_)‐mRNAs was reduced.^[^ ^80^ ^]^

Taken together, the results from various studies show again that the same modification can have different effects on the amount of protein produced, depending on the cell line or the transcript used.^[^ ^84^ ^]^

### 2.3 Immunogenicity

Uncapped RNA starts with a 5′ triphosphate (5′ ppp‐RNA) and is common for viral mRNAs. It is well‐established that 5′ caps play an important role in the discrimination between self and foreign mRNA.^[^ ^13^ ^,^ ^41^ ^,^ ^85^ ^]^ The 5′ cap decreases the intrinsic immunogenicity of mRNAs by preventing recognition of the 5′ triphosphate by pathogen recognition receptors (PRRs).^[^ ^86^ ^,^ ^87^ ^]^ This effect can already be observed for cap 0‐mRNAs. Cap 1 further reduces immunogenicity compared to cap 0, as it prevents the mRNA from recognition by PRRs.^[^ ^13^ ^,^ ^14^ ^,^ ^55^ ^,^ ^88^ ^,^ ^89^ ^]^ The most important PRRs known to interact directly with the 5′ cap are RIG‐1, and IFIT1 and IFIT5^[^ ^13^ ^,^ ^14^ ^,^ ^41^ ^]^ (Figure 1C).

RIG‐1 can bind both uncapped and cap 0‐mRNA but the interaction of RIG‐1 with capped mRNA is weaker than with 5′ triphosphate mRNA.^[^ ^87^ ^,^ ^90^ ^]^ RIG‐1 binding for cap 1‐mRNA is even further reduced.^[^ ^53^ ^]^ Another experiment—although not with mRNA—illustrates the importance of the methylation status of the first or second transcribed nucleotide regarding immunogenicity of mRNA. It was shown that chemically synthesized 10‐bp blunt‐ended hairpin RNAs (dsRNAs) containing cap 0 activate RIG‐1 in cells, similar to 5′ ppp‐dsRNA. In contrast, cap 1‐capped dsRNA resulted in strongly decreased RIG‐1‐activation. This effect can be explained by the reduced binding affinity of cap 1‐RNA (Kd = 425 nM) compared to cap 0 (Kd = 1.7 nM) and 5′ ppp‐RNA (Kd = 1.8 nM) to RIG‐1.^[^ ^13^ ^]^ A recent study found that cap 2 suppresses RIG‐1 activation and virus‐induced innate immune response. The binding affinity of RIG‐1 to cap 2 is markedly decreased in comparison to cap 1.^[^ ^53^ ^]^ Even in 5′ ppp‐RNA the methylation of only the second transcribed nucleotides (5′ pppNN_m_‐RNA) reduced IFN‐alpha production by 30% compared to unmethylated 5′ ppp‐RNA.^[^ ^91^ ^]^

The impact of the 2′‐*O*‐methylation at the TSN was also investigated in mice (containing a mutant 2′‐*O*‐methyltransferase active site), infected with the human coronavirus. The methylation reduced the recognition of viral mRNA in mice, preventing MDA5‐dependent production of type I interferons, thus leading to a lower activation of the immune system.^[^ ^92^ ^]^ Further methylation of the TSN (m^6^A_m_), however, showed no impact on mRNA immunogenicity in initial experiments using the reporter cell line HEK‐NF‐κB.^[^ ^83^ ^]^

While 5′ triphosphorylated mRNA is detected by both IFIT1 and IFIT5, only IFIT1 is able to detect cap 0‐mRNAs.^[^ ^14^ ^]^ Moreover, the 2′‐*O*‐methylation of the first or second transcribed nucleotide leads to a lower recognition (2′‐*O*‐methylation reduces binding and activation) through IFIT1.^[^ ^14^ ^,^ ^41^ ^]^ As it is known that IFIT1 competes with eIF4E for binding to the 5′ cap, one can hypothesize that a reduced recognition by IFIT1 is likely to correlate with increased translation.^[^ ^14^ ^,^ ^93^ ^]^

Taken together, in contrast to 5′ ppp‐mRNAs or cap 0‐mRNAs, cap 1 and especially cap 2 prevent recognition of the mRNA by PRRs and IFIT proteins.^[^ ^13^ ^,^ ^14^ ^,^ ^53^ ^]^

## 3 Non‐Natural 5′ Cap Modifications and Their Effect on Stability, Translation, and Immunogenicity

The ability to modulate specific features of mRNA is essential for its biotechnological and biomedical applications. Various 5′ cap modifications occur naturally and significantly influence the properties and interactions of mRNA. However, the use of natural modifications often presents constraints, primarily limited to methyl groups.^[^ ^47^ ^,^ ^94^ ^]^ To address these limitations, non‐natural modifications have been tested to expand the range of possibilities. These modifications can target the entire mRNA sequence, as well as the 3′ end and the 5′ cap. We will focus on the 5′ cap, which can be altered at the riboses, the nucleobases, or the 5′‐5′ triphosphate bridge. The diverse options for modifying the 5′ cap enable the precise optimization of mRNA properties, enhancing its utility in biomedical and biotechnological applications, and positioning mRNA modifications as a valuable tool in these fields.

**Figure 4:**
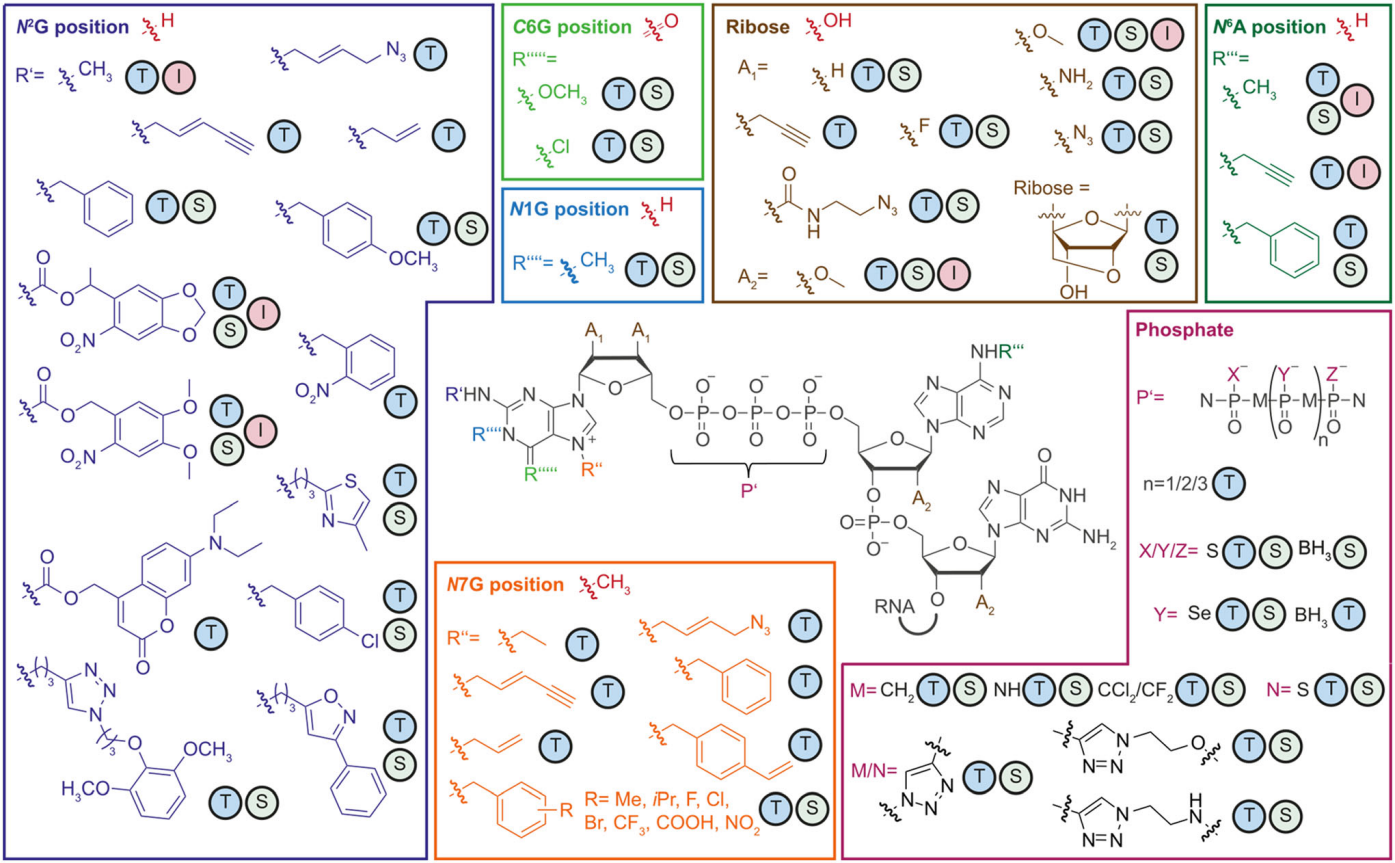
Overview of mRNA modifications at various positions of the 5′ cap. Central panel shows mRNA with modification options at the 5′ cap. Surrounding panels show modifications at individual positions. The substituent of the native cap 0 structure at every position is shown in red. Circled letters indicate which mRNA properties have been investigated for the respective modification. Translation (T), stability (S), and immunogenicity (I).

An overview of the natural and non‐natural 5′ cap modifications mentioned in this review is given in **Figure** **4**. The illustration also contains information on the tested mRNA properties.

**Figure 5:**
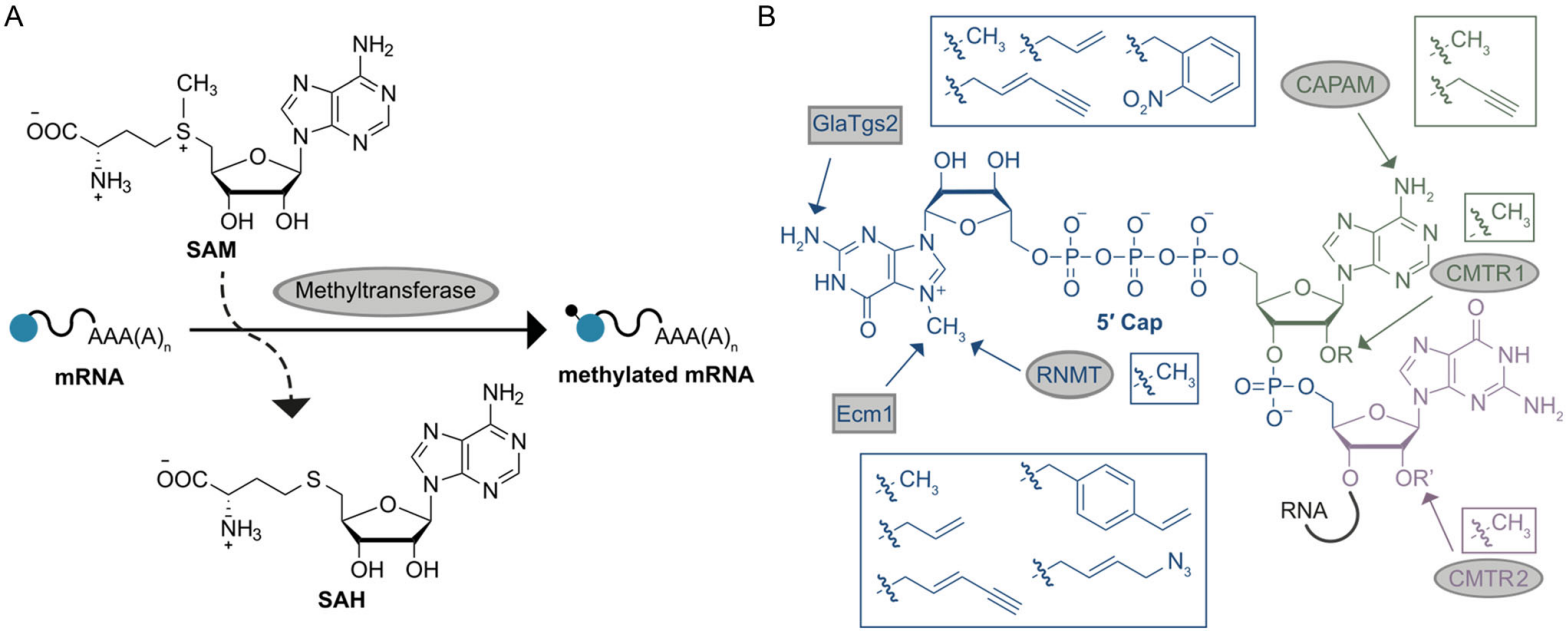
A) Modification of mRNA by methyltransferases. AdoMet‐dependent methyltransferases use AdoMet (SAM) as cofactor, forming AdoHcy (SAH) and the methylated mRNA. Besides methyl groups, other natural and non‐natural groups can be transferred by methyltransferases using the respective AdoMet analog. B) Modification of the 5′ cap by methyltransferases. RNMT, CAPAM, CMTR1, and CMTR2 are naturally occurring methyltransferases in mammals. In addition, GlaTgs2 and Ecm1 from foreign organisms can be used to transfer natural or non‐natural modifications to mammalian 5′ caps. Boxes indicate the natural and non‐natural modifications that have already been transferred by these enzymes and screened for mRNA properties.

Modifications of the 5′ cap can be introduced during its chemical synthesis or post‐synthetically by enzymes. While the chemical synthesis is almost unlimited with respect to the site and function of modifications, the post‐synthetic enzymatic modification relies on the natural methyltransferases (**Figure** **5**A). They methylate mRNA at various positions of the 5′ cap using AdoMet as co‐substrate and can sometimes use the dinucleotides GpppN or m^7^GpppN as minimal substrate.^[^ ^94^, ^95^, ^96^ ^]^ Thanks to their co‐substrate promiscuity or protein engineering, non‐natural groups can be enzymatically transferred from synthetic cofactor analogs.^[^ ^65^ ^]^ Thus, both natural and non‐natural modifications can be introduced enzymatically (Figure 5). In the following sections, we will discuss non‐natural 5′ cap modifications (either introduced chemically or enzymatically) that affect mRNA stability, translation, and immunogenicity classified by the position of modification.

### 3.1 Modification of the Ribose of m^7^G

Naturally occurring 5′ cap modifications impact mRNA properties, however, it can be challenging to get correctly capped mRNA. The dinucleotide m^7^GpppG can be incorporated up to 50% in the reverse orientation during IVT.^[^ ^97^ ^,^ ^98^ ^]^ This results in Gppp(m^7^G)‐mRNA that will not be translated.

The ARCA is (unnaturally) modified at the ribose of the m^7^G, preventing elongation in the case of wrong cap orientation. The 3′‐OH of the m^7^G can either be methylated or deoxygenated (m_2_^7,3′‐*O*^GpppG or m^7^3′dGpppG),^[^ ^97^ ^,^ ^99^ ^]^ however the commercially available analog refers to m_2_^7,3′‐*O*^GpppG. While deoxyribose‐ARCA‐mRNA yields similar translational output as cap 0‐mRNA, the translation of ribose‐ARCA‐capped mRNAs is enhanced in different mammalian cell lines (increase differs between cell lines) and rabbit reticulocyte lysate (RRL, 2.5‐fold increase).^[^ ^99^, ^100^, ^101^ ^]^ In addition, the stability of ARCA‐mRNAs was increased in MM3MG cells compared to cap 0‐mRNAs.^[^ ^100^ ^]^ Similar results regarding translation were found for methylation at the 2′‐OH.^[^ ^97^ ^]^ The binding affinity of ARCA‐mRNA to eIF4E was increased compared to cap 0‐mRNA. Furthermore, ARCA protected RNA from binding to IFIT1 to similar level to cap 1.^[^ ^102^ ^]^ In summary, ribose‐ARCAs allow for increased translational output due to correct incorporation, increased eIF4E affinity, and reduced immunogenicity. This has made the ARCA cap a standard cap since its development in 2003,^[^ ^97^ ^]^ before the cap 1 analogs became commercially available.

Additionally, mRNAs containing locked nucleic acids (LNA) at the 5′ cap (m^7(LNA)^GpppG) were produced via IVT. Two research groups report an increase in translational output in HeLa cells compared to cap 0 (3.2‐fold^[^ ^103^ ^]^ and 4.5‐fold,^[^ ^104^ ^]^ respectively). This effect can be explained by an enhanced eIF4E binding affinity and reduced susceptibility against hDcp2.^[^ ^104^ ^]^ An LNA‐containing cap 1 analog (m^7(LNA)^GpppA_m_G) led to a fivefold increase in GFP fluorescence compared to ARCA‐ or CleanCap‐mRNA in JAWSII cells.^[^ ^105^ ^]^

While the most common method for preparing capped mRNA in vitro is IVT in the presence of a cap analog, alternatively also post‐transcriptional enzymatic capping can be performed. After IVT, GTP can be transferred to the 5′ triphosphorylated mRNA in the presence of a capping enzyme. Recently, it was reported that a capping enzyme derived from the vaccinia virus capping enzyme (VCE) is able to react with different GTP analogs instead of natural GTP.^[^ ^106^ ^]^ In this study, almost all tested mRNAs, treated with ribose‐modified GTP analogs, were more susceptible to DcpS and Dcp2 than mRNA capped with unmodified GTP analog. Only the introduction of a propargyl group resulted in similar or even slightly increased stability compared to unmodified mRNA. However, this 2′‐*O*‐propargylated mRNA strongly decreased translational output in HeLa and HEK293 FT cells. The decreased translation might be explained by the low *N*7‐methylation levels for this modification when using VCE.^[^ ^106^ ^]^ In contrast, when installing a propargyl group to the 3′‐OH group of a trinucleotide cap analog (m^7,3′‐*O*‐propargyl^GpppA_m_G) via chemical synthesis, an increase in translational output was observed.^[^ ^107^ ^]^ Even though the use of VCE allows incorporation of several other groups than methyl at the ribose, only 2′‐OMe‐GTP (leading to ARCA‐mRNA) showed improved mRNA properties.

### 3.2 Modification of the Nucleobase

#### 3.2.1 *Modification of the* *N* *^2^* *Position of m* *^7^* *G*

Two different ways of modifying the *N* ^2^ position of m^7^G in ARCA have been investigated—chemical synthesis and enzymatic modification. Using the *Giardia lamblia* trimethylguanosinesynthase 2 (GlaTgs2) and AdoMet, a single methyl group can be transferred to the *N* ^2^ position producing 2,7‐dimethylguanosine (DMG), whose function is still unknown.^[^ ^108^ ^]^ The enzyme variant GlaTgs V34A was more promiscuous, permitting transfer of bigger residues than methyl, including benzyl residues and photocleavable ONB‐derivatives to the *N* ^2^ position of m^7^G (Figure 5B). The incorporation of natural and non‐natural groups at the *N* ^2^ position decreased translation in RRL and different cell lines, including HEK293T and HeLa cells.^[^ ^83^ ^,^ ^95^ ^,^ ^109^ ^]^ While the translational output of mRNAs modified at the *N* ^2^ position of m^7^G was decreased, methylation of the *N* ^2^ position did not impact mRNA immunogenicity in an NF‐κB responsive reporter cell line.^[^ ^83^ ^]^

Methyl groups at the *N* ^2^ position can also be installed chemically. To investigate the influence of further methylation at the *N* ^2^ position of cap 0 in mRNA, two dinucleotides were synthesized (di‐ (m_2_^2,7^GpppG) and tri‐ (m_3_^2,2,7^GpppG) methylated guanosine cap) and co‐transcriptionally incorporated into β‐globin‐mRNA.^[^ ^110^ ^]^ The 2,2,7‐trimethylguanosine (TMG) cap naturally occurs in eukaryotic snRNA and snoRNA.^[^ ^111^, ^112^, ^113^ ^]^ In contrast to the enzymatic installation of an additional methyl group at the *N* ^2^ position, incorporation of the synthesized dinucleotide (m_2_^2,7^GpppG) leads to an enhanced translation (1.5‐fold) compared to cap 0 in RRL.^[^ ^110^ ^]^ Trimethylated mRNA, containing the m_3_^2,2,7^GpppG cap, however decreased translational output (0.24‐fold) in RRL.^[^ ^110^ ^]^

Various benzylic groups on the *N* ^2^ position of m^7^G were also tested. Kocmik et al. used chemical synthesis and then co‐transcriptionally incorporated ARCAs with a benzyl‐ or 4‐methoxybenzyl group at the *N* ^2^ position into firefly luciferase‐mRNA. In comparison to cap 0, these caps resulted in a 2.4‐fold (4‐methoxybenzyl moiety) to 3.3‐fold (benzyl moiety) increase in translational output in HEK293 cells, even higher than the unmodified ARCA.^[^ ^114^ ^]^ Moreover, these capped mRNAs showed a higher susceptibility to Dcp1/2 after 5 min in comparison to ARCAs in vitro.^[^ ^114^ ^]^ Interestingly, after 48 h about 77% of the mRNA with a benzyl modification was still present in HEK293 cells, whereas incorporation of a 4‐methoxybenzyl group resulted in 55% remaining mRNA.^[^ ^114^ ^]^ Therefore, these modified mRNAs show similar or even increased stability compared to unmodified ARCAs (54% remaining mRNA after 48 h).^[^ ^114^ ^]^ Another study recently reported similar results when investigating the mRNA properties of chemically synthesized *N* ^2^‐modified dinucleotide cap analogs. These modified ARCAs (bn^2^m_2_^7,3′O^GpppG, (p‐OCH_3_bn)^2^m_2_^7,3′O^GpppG and bn^2^m_2_^7,2′O^GpppG) improved the translational output compared to the ARCA‐capped transcripts in both RRL and HEK293 cells, as wells as mRNA stability.^[^ ^115^ ^]^ This effect might be explained by a higher affinity to eIF4E of the modified mRNAs, which might correlate with increased stability due to limited access of decapping enzymes to the 5′ cap.

Taken together, these results show that modifications of the *N* ^2^ position of m^7^G impact translational output and stability. Especially introducing a benzylic modification at this position increases protein production and improves stability.

#### 3.2.2 *Replacing the Methyl Group of the m* *^7^* *G Nucleobase*

The key natural modification of the 5′ cap is the methylation at the *N*7 position of the added guanosine. The positive charge of m^7^G enhances its capacity for π‐stacking and fosters various cap‐specific interactions, such as its interaction with eIF4E.^[^ ^20^ ^,^ ^81^ ^]^ In humans, RNMT is the responsible methyltransferase, but recombinantly produced Ecm1 can be used to modify the *N*7 position and even install non‐natural groups efficiently in vitro^[^ ^65^ ^]^ on synthetic unmethylated 5′ caps (GpppN) or GpppG‐capped‐mRNAs. Ecm1, a methyltransferase from the parasite *Encephalitozoon cuniculi*, shows remarkable promiscuity and has been used to transfer various alkyl and benzylic groups^[^ ^65^ ^,^ ^95^ ^]^ (Figure 5B). Ecm1 was also used in a cascade reaction with a methionine adenosyltransferase, enabling the regioselective benzylation on GpppG‐capped mRNAs.^[^ ^116^ ^]^

As alternative to enzymatic modifications, chemically modified cap dinucleotides with ethyl and benzyl groups at the *N*7 position were prepared and used for co‐transcriptional capping of β‐globin‐mRNA.^[^ ^117^ ^]^ Moreover, Wojcik et al. chemically synthesized 17 novel bn^7^GpppG cap analogs that differ in polarity, size, and electron‐withdrawing properties. These analogs contained at least one methyl, isopropyl, halogen, trifluoromethyl, carboxyl, or nitro residue at the phenyl ring or were modified with a naphthalene moiety on the *N*7 position.^[^ ^118^ ^]^

The incorporation of a non‐natural alkyl group at the *N*7 position almost always decreases the affinity for eIF4E and reduces the amount of protein produced in vitro and in cells.^[^ ^95^ ^]^ For example, *N7* ethyl G inhibited protein production (0.75–fold relative to cap 0) in RRL^[^ ^117^ ^]^ and allyl had an even stronger negative effect in a different study in vitro and in cells.^[^ ^95^ ^]^

Interestingly, benzylic groups cause a less drastic reduction^[^ ^95^ ^,^ ^104^ ^]^ or even increase (e.g., 1.8‐fold^[^ ^117^ ^]^ or fivefold^[^ ^118^ ^]^) in translation. Similar effects were observed for most of the *N*7‐arylmethyl substitutions, only the 5′ cap carrying the *p*‐carboxybenyzl group decreased the translation.^[^ ^118^ ^]^ The highest protein production was found for the 5′ cap modified with a 4‐chlorobenzyl group on the phenyl ring, which resulted in 2.5‐fold higher protein production compared to ARCA.^[^ ^118^ ^]^ In particular, the 4‐chlorobenzyl group has consistently been reported to increase the amount of protein produced.^[^ ^116^ ^,^ ^118^ ^]^ Although the observed effects differ in quantity and for different cell lines, there is evidence in the literature that the benzylic‐*N*7G modifications of the 5′ cap can be leveraged to increase the amount of protein produced from a given transcript.

Furthermore, the *N*7‐benzyl group does not increase resistance to DcpS compared to m^7^GpppG.^[^ ^118^ ^]^ Additional substitutions, in contrast, increase stability. Especially bulkier groups on the phenyl ring such as isopropyl, trifluoromethyl, carboxyl, or the naphthalene group led to a higher resistance to DcpS (around 90%). Around 30% of the halogen‐containing dinucleotides were digested after 1 h.^[^ ^118^ ^]^

Taken together, several lines of evidence suggest that 4‐chlorobenzyl instead of methyl at the N7G of the 5′ cap increases the translational output of mRNAs, whereas alkyl groups have a negative impact.

#### 3.2.3 *Modification of Adenosine as TSN at Position* *N* *^6^*

The 5′ cap can also be altered by modifying the TSN, which is known to influence mRNA properties.^[^ ^55^ ^]^ When adenosine serves as the TSN, the methyltransferase CAPAM post‐transcriptionally modifies cap 1 at the *N* ^6^ position of TSN using AdoMet analogs with short alkyl chains, such as the propargyl group^[^ ^83^ ^]^ (Figure 5B). The resulting m^7^Gppp(prop^6^A_m_)‐mRNA is efficiently translated in HEK‐NF‐κB and HeLa cells, reaching a protein production similar to ARCA‐capped mRNAs (90%).^[^ ^83^ ^]^ In comparison to all other tested 5′ caps, including CleanCap and ARCA, this modification leads to a threefold higher immune response in HEK‐NF‐κB cells, although the reason for that was not yet elucidated.^[^ ^83^ ^]^

Recently, an *N* ^6^‐benzyl analog (m^7^Gppp^bn6^A_m_pG) was chemically synthesized.^[^ ^119^ ^]^ The so‐called AvantCap can be incorporated into mRNA via IVT. It showed improved translational output in CT26, primary murine and human dendritic cells, while no effect could be seen in HEK293T and A549 cells. When the AvantCap was tested in mice, it showed three‐ to sixfold higher protein production compared to m^7^GpppA_m_pG, depending on the mRNA and lipid nanoparticle formulation.^[^ ^119^ ^]^ The susceptibility of short RNA capped with the AvantCap to Dcp2 is slightly increased compared to m^7^GpppA_m_pG‐capped RNA.^[^ ^119^ ^]^

In summary, while small alkyl groups at the *N* ^6^ position of TSN seem to have little impact, the benzyl group appears to increase translational output. A moderately increased immune response was observed for the propargyl group. (**Table** **1**)

**Table 1:** Overview of the most effective non‐natural modifications at the different nucleobase positions presented in Section 3.2.

Modified position	Effect	Strongest effect
*N* ^2^ position of m^7^G	Impact on protein production and stability, no effect on immunogenicity reported	Benzyl group
*N7* position (replacing natural *N7* methylation)	Impact on protein production and stability, no effect on immunogenicity reported	4‐Chlorobenzyl group
*N* ^*6*^ position of transcription start nucleotide	Impact on translation and immune response	Benzyl group

**Table 2:** Overview over the most effective non‐natural modifications at the triphosphate bridge presented in Section 3.3.

Modified position	Effect	Strongest effect
Length of phosphate bridge	No significant improvement observed	–
Nonbridging oxygen atoms	Impact on translation and stability	β‐S‐ARCA
Bridging oxygen atoms	No significant improvement observed	–

### 3.3 Modifications in the Triphosphate Bridge of the 5′ Cap

**Figure 6:**
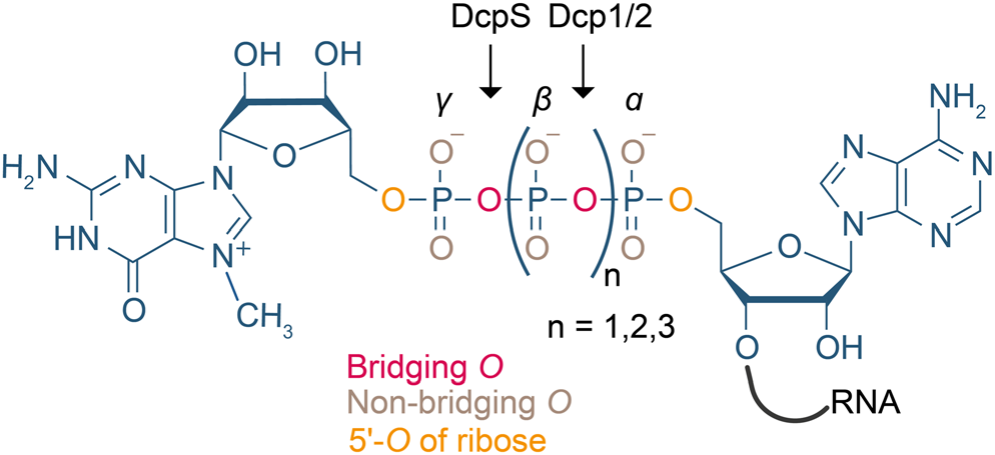
Modifications within the 5′‐5′ triphosphate bridge. Illustrated are the nonbridging (gray) as well as the bridging (red) oxygens within the triphosphate bridge and the 5′‐*O* of the ribose (orange). Moreover, additional phosphates were used to expand the bridge (indicated by n). Additionally, the cleavage sites of DcpS (between the γ‐ and β‐position) and Dcp1/2 (between the β‐ and α‐position) are illustrated.

In addition to modifications at the ribose or nucleobase, the 5′‐5′ triphosphate bridge can be modified. Decapping is one of the crucial steps in mRNA degradation. Dcp2 cleaves between the α‐ and β‐position,^[^ ^30^ ^,^ ^31^ ^]^ whereas DcpS cleaves between the β‐ and γ‐position.^[^ ^35^ ^,^ ^36^ ^]^ Hence, non‐natural modifications in the bridge can affect stability and translation (**Figure** **6**). Different lengths of the phosphate bridge and substitution of nonbridging and bridging oxygen atoms were tested with respect to effects on translation and stability. These have been reviewed in detail before.^[^ ^120^ ^,^ ^121^ ^]^

One 5′ cap analog that stands out is the β‐S‐ARCA, in which a nonbridging oxygen is replaced by a sulfur at the β‐position of the triphosphate bridge,^[^ ^122^ ^]^ significantly improving mRNA properties and even enabling its use in clinical trials (**Table** **2**).^[^ ^3^ ^]^

Jemielity et al. chemically synthesized 5′ caps with different lengths of the 5′‐5′ phosphate bridge and incorporated them into luciferase‐mRNA.^[^ ^97^ ^]^ For tri‐, tetra‐, and pentaphosphate (both for non‐ARCA and ARCA) 5′ caps the protein production in RRL was tested. Tetraphosphate caps slightly increased the translational output compared to their triphosphate counterparts, due to a higher affinity to eIF4E.^[^ ^22^ ^,^ ^97^ ^]^ In addition, a tetraphosphate cap with a benzyl group at the *N*7 position increased protein production (twofold) relative to cap 0, although it was similar to the translational output induced by the 5′ triphosphate cap with a benzylic modification at the *N*7 position.^[^ ^22^ ^]^ A 2.5‐fold increase in translational output was observed for m^2^bn^7^GppppG‐capped mRNA compared to cap 0.^[^ ^22^ ^]^

#### 3.3.1 *Modifications of the Non‐* *bridging Oxygen Atoms in the Triphosphate Bridge*

In addition to varying the length, the oxygen atoms of the 5′‐5′ bridge can be replaced by other atoms and groups (Figure 6). Substitution of non‐bridging oxygen atoms yielded ARCAs containing a phosphorothioate,^[^ ^123^ ^]^ phosphoroselenoate,^[^ ^124^ ^]^ or boranophosphate.^[^ ^125^ ^]^ For all of these 5′ caps, the stability and protein production of the resulting mRNAs were investigated. The mRNAs containing a sulfur at the α‐ or β‐position of the 5′ cap were resistant or partially resistant against degradation by Dcp2 in vitro.^[^ ^123^ ^]^ Of note, replacing oxygen by sulfur introduces a new stereocenter and the biochemical properties can vary for different stereoisomers. In HC11 cells, β‐S‐ARCA is significantly more stable than ARCA, whereas sulfur at the γ‐position did not influence mRNA stability, correlating to the in vitro findings.^[^ ^123^ ^]^ The decapping by Dcp2 could also be partially inhibited with a phosphoroselenoate or boranophosphate modification at the β‐position and is dependent on the stereochemistry.^[^ ^126^ ^]^ While analogs containing sulfur at the α‐ or β‐position were hydrolyzed by DcpS with similar efficiencies compared to the unmodified parent analog, sulfur analogs modified at the γ‐position were resistant to DcpS in vitro.^[^ ^23^ ^]^ Modification with boranophosphate decreased degradation through DcpS.^[^ ^125^ ^]^

Besides stability, also the impact on translation of mRNA with these modified 5′ caps was investigated. In comparison to ARCA, β‐S‐ARCA increased translational output in HC11 cells 2.4‐fold.^[^ ^123^ ^]^ One reason for this could be a more efficient recruitment of the 5′ cap to the polysomes and/or a higher binding affinity to eIF4E.^[^ ^23^ ^,^ ^123^ ^]^ The other 5′ caps resulted in protein production similar to ARCA.^[^ ^123^ ^]^ A slight enhancement of translation was also shown for mRNAs with ARCAs containing phosphoroselenoate at the β‐position in RRL^[^ ^124^ ^]^ and for mRNAs with ARCAs containing boranophosphate in dendritic cells.^[^ ^125^ ^]^ In HeLa cells, translation of 5′ caps modified with boranophosphate or selenophosphate depends on stereochemistry and varies between 0.5‐fold and 1.4‐fold compared to ARCA. This effect is smaller than for β‐S‐ARCA.^[^ ^126^ ^]^ Thus, β‐S‐ARCA increases the binding affinity to eIF4E, lowers the susceptibility to the decapping enzymes Dcp1/2, and enhances translation.^[^ ^123^ ^]^ Moreover, β‐S‐ARCA prevents binding of the transcript by IFIT1 protein similar to cap 1, which might be beneficial for therapeutic applications.^[^ ^102^ ^]^

Furthermore, the biological properties of 5′ caps with two sulfur atoms replacing the non‐bridging oxygen atoms in the triphosphate bridge were investigated. Depending on the stereochemistry, this can lead to higher stability due to lower degradation by Dcp1/2.^[^ ^127^ ^]^ In addition, cap‐dependent translation in dendritic cells can be increased compared to mRNA containing monophosphorothioate caps.^[^ ^127^ ^]^

#### 3.3.2 Modifications of the Bridging Oxygen Atoms in the Triphosphate Bridge

Similar studies were conducted for the bridging positions within the 5′‐5′ triphosphate bridge. The influence of different 5′ cap analogs, containing sulfur, a methylene group, a dihalogenmethylene group, or an imido group at bridging positions were tested on translation and stability. Incorporation of a methylene group between the α‐ and β‐position resulted in a slightly higher resistance against degradation by Dcp2 compared to ARCA.^[^ ^100^ ^]^ Capped mRNAs containing a sulfur at the 5′‐*O* position of one ribose (phosphorothiolate caps) (Figure 6) are less susceptible to degradation by DcpS and Dcp2, leading to a higher stability in vitro.^[^ ^128^ ^]^ In addition, incorporation of CCl_2_‐ or CF_2_‐substituted 5′ cap analogs leads to increased stability due to lower degradation by DcpS and Dcp2.^[^ ^129^ ^]^ Moreover, 5′ caps with a NH‐group in the cleavage site of DcpS were resistant to decapping by DcpS.^[^ ^130^ ^]^

In comparison to cap 0‐mRNA, the methylene containing 5′ cap led to a slightly higher translational output in MM3 MG cells, although this enhancement was smaller than for ARCA.^[^ ^100^ ^]^ Phosphorothiolate caps enhanced the translation 1.4‐fold compared to unmodified ARCA in HeLa cells.^[^ ^128^ ^]^ The translation of mRNAs containing NH‐groups in their 5′ cap was lower than for the original ARCA.^[^ ^130^ ^]^ CCl_2_‐substituted ARCAs resulted in similar translation as unmodified ARCAs in vitro.^[^ ^129^ ^]^

Furthermore, dinucleotide cap analogs with a phosphotriazole bridge were studied. To this end, two chemically synthesized mononucleotides or nucleoside analogs, containing an alkyne or azide moiety at the bridging position reacted via a CuAAC reaction.^[^ ^131^ ^]^ Most of the 5′ caps were more susceptible to Dcp2 than 2′‐ARCA. Only the RNA capped with m_2_^7,2′‐*O*^Gpp‐triazole‐C_2_H_4_OppG was very stable (94% intact mRNA after 1 h of incubation).^[^ ^131^ ^]^ To investigate the influence on translation in living HeLa cells, the dinucleotides were co‐transcriptionally incorporated into luciferase‐mRNA.^[^ ^131^ ^]^ All triazole modifications lowered protein production compared to ARCA‐mRNA when crude RNA (before digestion of uncapped mRNA after IVT) was used.^[^ ^131^ ^]^ After removal of uncapped mRNA, m_2_^7,2′‐*O*^Gppp‐triazole‐C_2_H_4_OpG‐mRNA resulted in slightly higher translational output than ARCA‐capped RNA.^[^ ^131^ ^]^ When a triazole modification is incorporated in tri‐ or tetranucleotide cap analogs, only mRNA carrying the m^7^Gppp‐triazole‐C_2_H_4_pA_m_pG cap led to comparable protein production in JAWS II cells as cap 1‐mRNA. Suceptibility to Dcp1/2 could be impaired when using a m^7^GpppCH_2_‐triazole‐A_m_pG cap.^[^ ^132^ ^]^

Taken together, modifications of the 5′‐5′ triphosphate bridge present an opportunity to increase translational output and stability, mainly based on lower susceptibility toward decapping enzymes. When comparing all modifications at the 5′‐5′ phosphate bridge, several modifications decrease susceptibility to decapping enzymes. However, only β‐S‐ARCA seems to additionally have a positive effect on protein production and is thus the most promising modification regarding the 5′‐5′ phosphate bridge (Table 2).

Recently, Chen et al. published data on mRNAs that contain two 5′ caps per mRNA.^[^ ^104^ ^]^ By incorporating 5‐octadiynyl‐dU into an mRNA strand and using CuAAC to attach a capped oligonucleotide with a 3′‐azide group, dual capped mRNAs were produced. An enhanced binding affinity of these mRNAs to eIF4E leads to enhanced translation efficiency.^[^ ^46^, ^59^, ^104^ ^]^ Together with other optimizing modifications in the UTRs, a dual‐LNAm^7^G‐LNA‐mRNA encoding for NLuc was injected into mice. This mRNA was highly expressed until 144 h post injection compared to 96 h for cap 0‐mRNA.^[^ ^104^ ^]^ Furthermore, circularized mRNAs were combined with a branched cap to create highly stable mRNAs (QRNAs) while maintaining efficient cap‐dependent translation initiation.^[^ ^104^ ^]^ Compared to circRNA with internal ribosome entry sites the QRNA led to sevenfold higher protein output. In mice, LNAm^7^G‐capped QRNA led to 59‐fold increased expression compared to the uncapped circRNA precursor after 48 h.^[^ ^104^ ^]^ Furthermore, a dual‐capped mRNA was tested for vaccine against SARS‐CoV‐2 and successfully induced a significantly stronger T helper cell immune response compared to cap 0‐mRNA.^[^ ^104^ ^]^

### 3.4 Emerging Applications of Modified 5′ Caps

The introduction of non‐natural groups provides a toolbox for new applications of the 5′ cap. For instance, click chemistry enables bioconjugation and several downstream applications. For click chemistry the 5′ caps require functional groups suitable for bioorthogonal and ideally nontoxic reactions. The copper‐catalyzed (CuAAC), strain‐promoted (SPAAC) azide‐alkyne click, and inverse electron‐demand Diels–Alder reactions have already been performed with RNA, primarily for internal and 3′ poly(A) tail modifications.^[^ ^133^, ^134^, ^135^ ^]^

**Figure 7:**
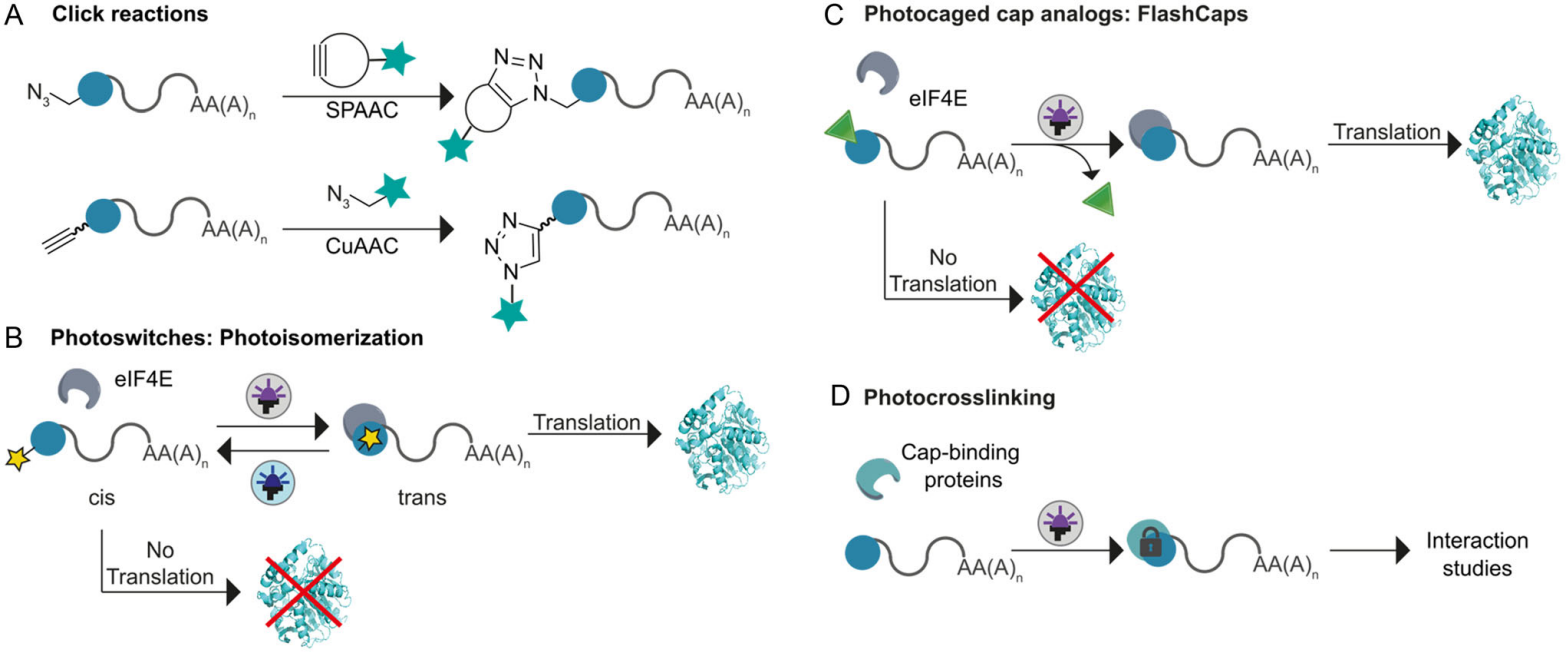
Schematic illustration of further utilization of 5′ caps. A) Click reactions at the 5′ cap allow mRNA labeling.^[^ ^94^ ^,^ ^95^ ^,^ ^106^, ^107^, ^108^ ^,^ ^136^ ^,^ ^137^ ^]^ B) Photoswitches enable control over translation via light using photoisomerization.^[^ ^140^ ^,^ ^141^ ^]^ C) The photocaged 5′ cap analogs, known as FlashCaps, enable the light‐mediated activation of translation without remaining alterations of the mRNA.^[^ ^142^ ^,^ ^145^ ^]^ D) 5′ Cap analogs containing photoactivatable groups can be used to study interaction with cap‐binding proteins.^[^ ^149^ ^]^

Evidently, also the 5′ cap can be conjugated by click chemistry (**Figure** **7**A). Clickable ARCAs were synthesized and incorporated into mRNA by IVT. The mRNA was translated in cells and modified via SPAAC.^[^ ^136^ ^]^ These 5′ cap analogs contained an azido group at the 2′‐ or 3′‐OH position of the m^7^G while the 5′‐5′ bridge remained either unmodified or contained a sulfur at the nonbridging β‐position or a tetraphosphate.^[^ ^136^ ^]^ All 5′ caps reduced the degradation of short RNA by Dcp2. As expected (based on the findings of Grudzien‐Nogalska^[^ ^123^ ^]^) RNAs containing a phosphorothioate in the 5′ cap was most stabilized (three‐ to 15‐fold lower susceptibility to decapping compared to cap 0 RNA).^[^ ^136^ ^]^ In HeLa cell lysate, the protein production of modified mRNAs was three‐ to eightfold higher than cap 0‐capped mRNA.^[^ ^136^ ^]^ Labeling of 3′‐N_3_‐m^7^Gpp_S_pG‐capped mRNA with Cy5 in vitro resulted in similar translational output compared to ARCA‐capped mRNAs in HeLa cells.^[^ ^136^ ^]^ Since SPAAC reaction is not toxic for cells, labeling was also carried out in living HeLa cells but resulted in lower efficiency compared to in vitro labeling.^[^ ^136^ ^]^ Furthermore, clicking was performed after introduction of azide‐containing GTP analogs that were transferred to mRNA with VCE. Subsequent fluorescent labeling with SPAAC resulted in a comparable amount of protein produced in HeLa cells as for mRNA treated with unmodified GTP analogs.^[^ ^106^ ^]^

Besides azides, a propargyl group was installed at the 3′‐OH group of a trinucleotide cap analog (m^7,3‐*O*‐propargyl^GpppA_m_G) via chemical synthesis.^[^ ^107^ ^]^ This non‐natural modification resulted in a 1.3‐fold increase in translational output in A549 cells relative to its unmodified counterpart (m^7^GpppA_m_G). Subsequent fluorescent labeling of the mRNA using click chemistry resulted still in a translationally active mRNA, especially after HPLC purification.^[^ ^107^ ^]^

Another option to further shape 5′ caps using click chemistry was realized by chemo‐enzymatic modification, in particular using methyltransferases and AdoMet analogs. Ecm1 was used to transfer allyl‐, pentenynyl‐, vinylbenzyl‐, or azidobutenyl groups to the *N*7G position of the 5′ cap by providing the respective AdoMet analogs.^[^ ^65^ ^,^ ^95^ ^]^ These modifications can be used for site‐specific labeling in subsequent click reactions with commercially available fluorophores in living HeLa cells.^[^ ^95^ ^]^ Complementary, GlaTgs2 was found to be able to transfer a propylene group to the *N* ^2^ position of cap 0 when the appropriate AdoMet analog is provided.^[^ ^137^ ^]^ The enzyme variant GlaTgs2 V34A allowed the transfer of allyl‐, pentenynyl‐, or azido‐butenyl groups using respective AdoMet analogs.^[^ ^94^ ^,^ ^137^ ^]^ These modifications were used to click a fluorophore via CuAAC or SPAAC reaction to capped RNAs.^[^ ^94^ ^,^ ^137^ ^]^ The allyl‐modified capped mRNA was moderately translated in RRL and living HeLa cells.^[^ ^95^ ^]^ GlaTgs2 V34A was further used to incorporate the aromatic group 4‐vinylbenzyl, at the mRNA 5′ cap by providing the appropriate AdoMet analog.^[^ ^108^ ^]^ This group can be used for bioorthogonal photoclick reactions, resulting in a fluorescent product applicable in mRNA imaging.^[^ ^108^ ^]^

Besides click chemistry, non‐natural modifications have been exploited to control mRNA properties. However, only a few approaches have been described to control mRNA properties by modifying the 5′ cap. In 2023, cap analogs, called ‘PureCap’, containing a hydrophobic tag at the ribose of the 5′ cap guanosine were produced. These tags can be used to separate uncapped and capped mRNA via HPLC purification, thus avoiding enzymatic treatment of the RNAs while improving immunogenetic properties. Upon irradiation by light, the tag is removed and the unmodified mRNAs can be used for transfection and other applications.^[^ ^138^ ^]^ Meng et al. also produced 2′‐deoxy‐modified PureCap analogs. In HeLa cells, the analog containing this modification at the ribose of the TSN showed a 3.2‐fold increased translational output compared to the standard cap 1‐type PureCap.^[^ ^139^ ^]^

Another concept allows the control of translation via *cis‐trans* photoisomerization in both mammalian cells and zebrafish embryos. The blocked translation is based on a lack of interaction of the 5′ cap to eIF4E in the *cis* state, while *trans* isomerization enables efficient translation^[^ ^140^ ^,^ ^141^ ^]^ (Figure 7B). However, in this approach, the mRNA remains altered. An alternative concept for light‐mediated activation of translation releases unaltered mRNA, that is, with a native cap 0. FlashCaps are 5′ cap analogs with a single photocageing group connected via a self‐immolative carbamate linkage at the *N* ^2^ position of cap 0 and can be used to regulate the expression of any given mRNA.^[^ ^142^ ^,^ ^143^ ^]^ FlashCaps prevent the mRNA from decapping by Dcp1/2, but are comparably stable *in cellula* (relative to cap 0‐mRNA). FlashCap‐mRNAs are barely translated in mammalian cells before irradiation, however, upon irradiation by light the amount of protein produced is similar as for cap 0‐capped mRNA.^[^ ^142^ ^]^ Again, the muted translation before irradiation is based on a lack of interaction with eIF4E and the FlashCaps (Figure 7C). Moreover, FlashCap‐mRNAs show no increased immunogenicity in comparison to cap 0‐mRNA in an NF‐ĸB responsive assay^[^ ^142^ ^]^ and were also functional in living zebrafish embryos.^[^ ^144^ ^]^ A coumarin‐based FlashCap, which allows photo‐deprotection at higher wavelength (450 nm instead of 365 nm) also enables light‐mediated activation of translation *in cellula*.^[^ ^145^ ^]^ The same photocleavable protection group was installed at the *N*7G of the 5′ cap to mute mRNA translation. Irradiation by light releases the unmethylated 5′ cap analog that can be methylated in vitro and in the cytoplasm for subsequent translation.^[^ ^146^ ^]^ Additional incorporation of a methylene group between the β‐ and γ‐position of the FlashCap leads to the Medronate‐FlashCap. This cap was shown to decrease the susceptibility to DcpS and increased stability in cell lysate compared to cap 0 and unmodified FlashCaps, even after irradiation by light.^[^ ^147^ ^]^ Combining light‐mediated activation with a tetraphosphate bridge can be used to investigate the interactions of tetraphosphate 5′ caps and cap‐binding or ‐modifying enzymes.^[^ ^148^ ^]^

Furthermore, 5′ cap analogs containing photoactivatable groups at either the m^7^G ribose, 2′‐*O*‐ribose of the TSN (A) or *N* ^6^‐position of the TSN (A) were synthesized and incorporated via IVT.^[^ ^149^ ^]^ After irradiation at 365 nm these caps were crosslinked to cap‐binding proteins, such as eIF4E and DcpS, which makes them suitable candidates to study complex mRNA‐protein interactions^[^ ^149^ ^]^ (Figure 7D).

These findings show that mRNA 5′ caps are not only useful for modifying and optimizing mRNA properties but can also be used for a variety of downstream applications, further expanding the usage and importance of mRNA modifications.

## 4 Summary and Perspectives

In this article, we highlighted important mRNA 5′ cap modifications that have been reported to impact mRNA stability, translation, and immunogenicity. A lot of valuable research has been done on mRNA capping, especially in the last two decades. It became obvious that mRNA properties can be influenced and optimized by various modifications. In addition, the 5′ cap can be used for other applications, such as fluorescent labeling. However, the impact not only on the cellular level but also on the human body needs to be further investigated. It has been shown several times that mRNA 5′ cap modifications can have different effects depending on the cell line. Therefore, it is important to learn more about how exactly individual modifications impact stability, translation, and also immunogenicity. Furthermore, the desired properties vary for different medical or biotechnological applications. Accordingly, the view should remain open for a wide variety of effects that the modifications exert on the mRNA. To further improve our understanding of mRNAs, rare or non‐natural cap analogs and their functions also need to be investigated. This will expand the possibilities to influence, optimize and control mRNA properties.

## Conflict of Interest

The authors declare no conflict of interest.

## References

[1] S. E. Oliver , J. W. Gargano , M. Marin , M. Wallace , K. G. Curran , M. Chamberland , N. McClung , D. Campos‐Outcalt , R. L. Morgan , S. Mbaeyi , J. R. Romero , H. K. Talbot , G. M. Lee , B. P. Bell , K. Dooling , Morb. Mortal. Wkly. Rep. 2020, 69, 1922. 10.15585/mmwr.mm6950e2 PMC7745957 33332292

[2] S. E. Oliver , J. W. Gargano , M. Marin , M. Wallace , K. G. Curran , M. Chamberland , N. McClung , D. Campos‐Outcalt , R. L. Morgan , S. Mbaeyi , J. R. Romero , H. K. Talbot , G. M. Lee , B. P. Bell , K. Dooling , Morb. Mortal. Wkly. Rep. 2021, 69, 1653. 10.15585/mmwr.mm695152e1 PMC9191904 33382675

[3] U. Sahin , E. Derhovanessian , M. Miller , B. P. Kloke , P. Simon , M. Löwer , V. Bukur , A. D. Tadmor , U. Luxemburger , B. Schrörs , T. Omokoko , M. Vormehr , C. Albrecht , A. Paruzynski , A. N. Kuhn , J. Buck , S. Heesch , K. H. Schreeb , F. Müller , I. Ortseifer , I. Vogler , E. Godehardt , S. Attig , R. Rae , A. Breitkreuz , C. Tolliver , M. Suchan , G. Martic , A. Hohberger , P. Sorn , et al., Nature 2017, 547, 222. 28678784 10.1038/nature23003

[4] M. van Dülmen , A. Rentmeister , Biochemistry 2020, 59, 1650. 32298088 10.1021/acs.biochem.0c00181

[5] T. Vavilis , E. Stamoula , A. Ainatzoglou , A. Sachinidis , M. Lamprinou , I. Dardalas , I. S. Vizirianakis , Pharmaceutics 2023, 15, 166. 36678793 10.3390/pharmaceutics15010166 PMC9866414

[6] K. Karikó , M. Buckstein , H. P. Ni , D. Weissman , Immunity 2005, 23, 165. 16111635 10.1016/j.immuni.2005.06.008

[7] K. Karikó , H. Muramatsu , F. Welsh , J. Ludwig , H. Kato , S. Akira , D. Weissman , Molecular Therapy 2008, 16, 1833. 18797453 10.1038/mt.2008.200 PMC2775451

[8] B. R. Anderson , H. Muramatsu , S. R. Nallagatla , P. C. Bevilacqua , L. H. Sansing , D. Weissman , K. Karikó , Nucleic Acids Res. 2010, 38, 5884. 20457754 10.1093/nar/gkq347 PMC2943593

[9] C. M. Wei , B. Moss , Proc. Natl. Acad. Sci. U.S.A. 1975, 72, 318. 164018 10.1073/pnas.72.1.318 PMC432296

[10] Y. Furuichi , K.‐I. Miura , Nature 1975, 253, 374. 163011 10.1038/253374a0

[11] Y. Furuichi , M. Morgan , S. Muthukrishnan , A. J. Shatkin , Proc. Natl. Acad. Sci. U.S.A. 1975, 72, 362. 1054511 10.1073/pnas.72.1.362 PMC432305

[12] N. Sonenberg , H. Trachsel , S. Hecht , A. J. Shatkin , Nature 1980, 285, 331. 6246452 10.1038/285331a0

[13] S. C. Devarkar , C. Wang , M. T. Miller , A. Ramanathan , F. Jiang , A. G. Khan , S. S. Patel , J. Marcotrigiano , Proc. Natl. Acad. Sci. U.S.A. 2016, 113, 596. 26733676 10.1073/pnas.1515152113 PMC4725518

[14] Y. M. Abbas , B. T. Laudenbach , S. Martínez‐Montero , R. Cencic , M. Habjan , A. Pichlmair , M. J. Damha , J. Pelletier , B. Nagar , Proc. Natl. Acad. Sci. U.S.A. 2017, 114, E2106. 28251928 10.1073/pnas.1612444114 PMC5358387

[15] M. Arribas‐Layton , D. Wu , J. Lykke‐Andersen , H. Song , Biochim. Biophys. Acta, Gene Regul. Mech. 2013, 1829, 580. 10.1016/j.bbagrm.2012.12.006 PMC3660425 23287066

[16] S. Muthukrishnan , G. W. Both , Y. Furuichi , A. J. Shatkin , Nature 1975, 255, 33. 165427 10.1038/255033a0

[17] J. D. Lewis , E. Izaurralde , Eur. J. Biochem. 1997, 247, 461. 9266685 10.1111/j.1432-1033.1997.00461.x

[18] G. Varani , Structure 1997, 5, 855. 9261078 10.1016/s0969-2126(97)00239-6

[19] N. Sonenberg , A. G. Hinnebusch , Cell 2009, 136, 731. 19239892 10.1016/j.cell.2009.01.042 PMC3610329

[20] I. Topisirovic , Y. V. Svitkin , N. Sonenberg , A. J. Shatkin , Wiley Interdiscip. Rev.: RNA 2011, 2, 277. 21957010 10.1002/wrna.52

[21] A. C. Gingras , B. Raught , N. Sonenberg , Annu. Rev. Biochem. 1999, 68, 913. 10872469 10.1146/annurev.biochem.68.1.913

[22] E. Grudzien , J. Stepinski , M. Jankowska‐Anyszka , R. Stolarski , E. Darzynkiewicz , R. E. Rhoads , RNA 2004, 10, 1479. 15317978 10.1261/rna.7380904 PMC1370634

[23] J. Kowalska , M. Lewdorowicz , J. Zuberek , E. Grudzien‐Nogalska , E. Bojarska , J. Stepinski , R. E. Rhoads , E. Darzynkiewicz , R. E. Davis , J. Jemielity , RNA 2008, 14, 1119. 18430890 10.1261/rna.990208 PMC2390807

[24] R. H. Singer , S. Penman , J. Mol. Biol. 1973, 78, 321. 4747634 10.1016/0022-2836(73)90119-8

[25] A. B. Shyu , M. E. Greenberg , J. G. Belasco , Genes Dev. 1989, 3, 60. 2496006 10.1101/gad.3.1.60

[26] A. Krowczynska , R. Yenofsky , G. Brawerman , J. Mol. Biol. 1985, 181, 231. 3856689 10.1016/0022-2836(85)90087-7

[27] W. P. Donovan , S. R. Kushner , Nucleic Acids Res. 1983, 11, 265. 6338477 10.1093/nar/11.2.265 PMC325713

[28] R. Baumeister , P. Flache , Ö. Melefors , A. v. Gabain , W. Hillen , Nucleic Acids Res. 1991, 19, 4595. 1653948 10.1093/nar/19.17.4595 PMC328697

[29] S. A. Emory , P. Bouvent , J. G. Belasco , Genes Dev. 1992, 6, 135. 1370426 10.1101/gad.6.1.135

[30] Z. R. Wang , X. Jiao , A. Carr‐Schmid , M. Kiledjian , Proc. Natl. Acad. Sci. U.S.A. 2002, 99, 12663. 12218187 10.1073/pnas.192445599 PMC130517

[31] M.‐G. Song , Y. Li , M. Kiledjian , Mol. Cell 2010, 40, 423. 21070968 10.1016/j.molcel.2010.10.010 PMC2982215

[32] D. Muhlrad , C. J. Decker , R. Parker , Genes Dev. 1994, 8, 855. 7926773 10.1101/gad.8.7.855

[33] J. H. Chang , X. Jiao , K. Chiba , C. Oh , C. E. Martin , M. Kiledjian , L. Tong , Nat. Struct. Mol. Biol. 2012, 19, 1011. 22961381 10.1038/nsmb.2381 PMC3711404

[34] X. Jiao , J. H. Chang , T. Kilic , L. Tong , M. Kiledjian , Mol. Cell 2013, 50, 104. 23523372 10.1016/j.molcel.2013.02.017 PMC3630477

[35] Z. R. Wang , M. Kiledjian , Cell 2001, 107, 751. 11747811 10.1016/s0092-8674(01)00592-x

[36] Y. Li , M. Kiledjian , Wiley Interdiscip. Rev.: RNA 2010, 1, 253. 21935889 10.1002/wrna.15 PMC13110874

[37] D. Y. Li , M. H. Wu , Signal Transduction Targeted Ther. 2021, 6, 24. 10.1038/s41392-020-00422-1 PMC7815747 33468999

[38] H. Kato , O. Takeuchi , E. Mikamo‐Satoh , R. Hirai , T. Kawai , K. Matsushita , A. Hiiragi , T. S. Dermody , T. Fujita , S. Akira , J. Exp. Med. 2008, 205, 1601. 18591409 10.1084/jem.20080091 PMC2442638

[39] H. Kato , O. Takeuchi , S. Sato , M. Yoneyama , M. Yamamoto , K. Matsui , S. Uematsu , A. Jung , T. Kawai , K. J. Ishii , O. Yamaguchi , K. Otsu , T. Tsujimura , C. S. Koh , C. R. E. Sousa , Y. Matsuura , T. Fujita , S. Akira , Nature 2006, 441, 101. 16625202 10.1038/nature04734

[40] A. Pichlmair , O. Schulz , C. P. Tan , J. Rehwinkel , H. Kato , O. Takeuchi , S. Akira , M. Way , G. Schiavo , C. R. E. Sousa , J. Virol. 2009, 83, 10761. 19656871 10.1128/JVI.00770-09 PMC2753146

[41] S. Daffis , K. J. Szretter , J. Schriewer , J. Li , S. Youn , J. Errett , T. Y. Lin , S. Schneller , R. Zust , H. Dong , V. Thiel , G. C. Sen , V. Fensterl , W. B. Klimstra , T. C. Pierson , R. M. Buller , M. Gale , P. Y. Shi , M. S. Diamond , Nature 2010, 468, 452. 21085181 10.1038/nature09489 PMC3058805

[42] A. J. Shatkin , J. L. Manley , Nat. Struct. Biol. 2000, 7, 838. 11017188 10.1038/79583

[43] A. Ramanathan , G. B. Robb , S. H. Chan , Nucleic Acids Res. 2016, 44, 7511. 27317694 10.1093/nar/gkw551 PMC5027499

[44] C. Fabrega , S. Hausmann , V. Shen , S. Shuman , C. D. Lima , Mol. Cell 2004, 13, 77. 14731396 10.1016/s1097-2765(03)00522-7

[45] A. J. Shatkin , Cell 1976, 9, 645. 1017010 10.1016/0092-8674(76)90128-8

[46] S. Shuman , W. E. Cohn , K. M. in , in Prog. Nucleic Acid Res. Mol. Biol, vol. 50, Academic Press, London, UK 1995, pp. 101–129. 7754031 10.1016/s0079-6603(08)60812-0

[47] F. Bélanger , J. Stepinski , E. Darzynkiewicz , J. Pelletier , J. Biol. Chem. 2010, 285, 33037. 20713356 10.1074/jbc.M110.155283 PMC2963352

[48] C.‐M. Wei , A. Gershowitz , B. Moss , Nature 1975, 257, 251. 1161029 10.1038/257251a0

[49] Y. Furuichi , M. Morgan , A. J. Shatkin , W. Jelinek , M. Salditt‐Georgieff , J. Darnell , Proc. Natl. Acad. Sci. U.S.A. 1975, 72, 1904. 1057180 10.1073/pnas.72.5.1904 PMC432656

[50] M. Werner , E. Purta , K. H. Kaminska , I. A. Cymerman , D. A. Campbell , B. Mittra , J. R. Zamudio , N. R. Sturm , J. Jaworski , J. M. Bujnicki , Nucleic Acids Res. 2011, 39, 4756. 21310715 10.1093/nar/gkr038 PMC3113572

[51] J. Wang , B. L. A. Chew , Y. Lai , H. Dong , L. Xu , S. Balamkundu , W. M. Cai , L. Cui , C. F. Liu , X. Y. Fu , Z. Lin , P. Y. Shi , T. K. Lu , D. Luo , S. R. Jaffrey , P. C. Dedon , Nucleic Acids Res. 2019, 47, e130. 31504804 10.1093/nar/gkz751 PMC6847653

[52] A. Galloway , V. H. Cowling , Biochim. Biophys. Acta, Gene Regul. Mech. 2019, 1862, 270. 30312682 10.1016/j.bbagrm.2018.09.011 PMC6414751

[53] V. Despic , S. R. Jaffrey , Nature 2023, 614, 358. 36725932 10.1038/s41586-022-05668-z PMC9891201

[54] S. Akichika , S. Hirano , Y. Shichino , T. Suzuki , H. Nishimasu , R. Ishitani , A. Sugita , Y. Hirose , S. Iwasaki , O. Nureki , T. Suzuki , Science 2019, 363, 141. 10.1126/science.aav0080 30467178

[55] P. J. Sikorski , M. Warminski , D. Kubacka , T. Ratajczak , D. Nowis , J. Kowalska , J. Jemielity , Nucleic Acids Res. 2020, 48, 1607. 31984425 10.1093/nar/gkaa032 PMC7038993

[56] D. R. Schoenberg , L. E. Maquat , Trends Biochem. Sci. 2009, 34, 435. 19729311 10.1016/j.tibs.2009.05.003 PMC2743798

[57] Y. Otsuka , N. Kedersha , D. Schoenberg , Mol. Cell. Biol. 2009, 29, 2155. 19223470 10.1128/MCB.01325-08 PMC2663312

[58] E. Grudzien‐Nogalska , J. G. Bird , B. E. Nickels , M. Kiledjian , RNA 2018, 24, 1418. 30045887 10.1261/rna.067686.118 PMC6140466

[59] M. Wolfram‐Schauerte , N. Pozhydaieva , J. Grawenhoff , L. Welp , I. Silbern , A. Wulf , F. Billau , T. Glatter , H. Urlaub , A. Jäschke , K. Höfer , Nature 2023, 620, 1054. 37587340 10.1038/s41586-023-06429-2 PMC10468400

[60] J. M. Henderson , A. Ujita , E. Hill , S. Yousif‐Rosales , C. Smith , N. Ko , T. McReynolds , C. R. Cabral , J. R. Escamilla‐Powers , M. E. Houston , Curr. Protoc. 2021, 1, e39. 33524237 10.1002/cpz1.39

[61] U. Sahin , A. Muik , E. Derhovanessian , I. Vogler , L. M. Kranz , M. Vormehr , A. Baum , K. Pascal , J. Quandt , D. Maurus , S. Brachtendorf , V. Lörks , J. Sikorski , R. Hilker , D. Becker , A.‐K. Eller , J. Grützner , C. Boesler , C. Rosenbaum , M.‐C. Kühnle , U. Luxemburger , A. Kemmer‐Brück , D. Langer , M. Bexon , S. Bolte , K. Karikó , T. Palanche , B. Fischer , A. Schultz , P.‐Y. Shi , et al., Nature 2020, 586, 594. 32998157 10.1038/s41586-020-2814-7

[62] L. Miao , Y. Zhang , L. Huang , Mol. Cancer 2021, 20, 23. 33632261 10.1186/s12943-021-01335-5 PMC7905014

[63] K. S. Corbett , D. K. Edwards , S. R. Leist , O. M. Abiona , S. Boyoglu‐Barnum , R. A. Gillespie , S. Himansu , A. Schäfer , C. T. Ziwawo , A. T. DiPiazza , K. H. Dinnon , S. M. Elbashir , C. A. Shaw , A. Woods , E. J. Fritch , D. R. Martinez , K. W. Bock , M. Minai , B. M. Nagata , G. B. Hutchinson , K. Wu , C. Henry , K. Bahl , D. Garcia‐Dominguez , L. Ma , I. Renzi , W.‐P. Kong , S. D. Schmidt , L. Wang , Y. Zhang , et al., Nature 2020, 586, 567. 32756549 10.1038/s41586-020-2622-0 PMC7581537

[64] F. Muttach , N. Muthmann , A. Rentmeister , Beilstein J. Org. Chem. 2017, 13, 2819. 30018667 10.3762/bjoc.13.274 PMC5753152

[65] A. Bollu , A. Peters , A. Rentmeister , Acc. Chem. Res. 2022, 55, 1249. 35420432 10.1021/acs.accounts.2c00059

[66] M. Fink , G. Flekna , A. Ludwig , T. Heimbucher , T. Czerny , Dev. Dyn. 2006, 235, 3370. 17068769 10.1002/dvdy.20995

[67] K. Leppek , G. W. Byeon , W. Kladwang , H. K. Wayment‐Steele , C. H. Kerr , A. D. F. Xu , D. Kim , V. V. Topkar , C. Choe , D. Rothschild , G. C. Tiu , R. Wellington‐Oguri , K. Fujii , E. Sharma , A. M. Watkins , J. J. Nicol , J. Romano , B. Tunguz , F. Diaz , H. Cai , P. B. Guo , J. W. Wu , F. Y. Meng , S. Shi , E. Participants , P. R. Dormitzer , A. Solorzano , M. Barna , R. Das , Nat. Commun. 2022, 13, 22. 35318324 10.1038/s41467-022-28776-w PMC8940940

[68] S. Linares‐Fernandez , C. Lacroix , J. Y. Exposito , B. Verrier , Trends Mol. Med. 2020, 26, 311. 31699497 10.1016/j.molmed.2019.10.002

[69] D. M. Mauger , B. J. Cabral , V. Presnyak , S. V. Su , D. W. Reid , B. Goodman , K. Link , N. Khatwani , J. Reynders , M. J. Moore , I. J. McFadyen , Proc. Natl. Acad. Sci. U.S.A. 2019, 116, 24075. 31712433 10.1073/pnas.1908052116 PMC6883848

[70] F. Hia , S. Yang , Y. Shichino , M. Yoshinaga , Y. Murakawa , A. Vandenbon , A. Fukao , T. Fujiwara , M. Landthaler , T. Natsume , S. Adachi , S. Iwasaki , O. Takeuchi , EMBO Rep. 2019, 20, EMBR201948220. 10.15252/embr.201948220 PMC6831995 31482640

[71] S. C. Kim , S. S. Sekhon , W. R. Shin , G. Ahn , B. K. Cho , J. Y. Ahn , Y. H. Kim , Mol. Cell. Toxicol. 2022, 18, 1. 34567201 10.1007/s13273-021-00171-4 PMC8450916

[72] Q. S. Wu , A. A. Bazzini , Annu. Rev. Biochem. 2023, 92, 227. 37001134 10.1146/annurev-biochem-052621-091808

[73] M. Warminski , A. Mamot , A. Depaix , J. Kowalska , J. Jemielity , Acc. Chem. Res. 2023, 56, 2814. 37782471 10.1021/acs.accounts.3c00442 PMC10586375

[74] F. Picard‐Jean , C. Brand , M. Tremblay‐Létourneau , A. Allaire , M. C. Beaudoin , S. Boudreault , C. Duval , J. Rainville‐Sirois , F. Robert , J. Pelletier , B. J. Geiss , M. Bisaillon , PLoS ONE 2018, 13, e0193804. 29601584 10.1371/journal.pone.0193804 PMC5877831

[75] K. Drazkowska , N. Baran , M. Warminski , R. Tomecki , A. Depaix , D. Cysewski , R. Kasprzyk , J. Kowalska , J. Jemielity , P. J. Sikorski , Nucleic Acids Res. 2022, 50, 9051. 36018811 10.1093/nar/gkac722 PMC9458431

[76] E. Grudzien‐Nogalska , M. Kiledjian , Wiley Interdiscip. Rev.: RNA 2017, 8, e1379. 10.1002/wrna.1379 PMC5179306 27425147

[77] J. Mauer , X. B. Luo , A. Blanjoie , X. Jiao , A. V. Grozhik , D. P. Patil , B. Linder , B. F. Pickering , J. J. Vasseur , Q. Chen , S. S. Gross , O. Elemento , F. Debart , M. Kiledjian , S. R. Jaffrey , Nature 2017, 541, 371. 28002401 10.1038/nature21022 PMC5513158

[78] K. Boulias , D. Toczydlowska‐Socha , B. R. Hawley , N. Liberman , K. Takashima , S. Zaccara , T. Guez , J. J. Vasseur , F. Debart , L. Aravind , S. R. Jaffrey , E. L. Greer , Mol. Cell 2019, 75, 631. 31279658 10.1016/j.molcel.2019.06.006 PMC6703822

[79] J. Wei , F. Liu , Z. Lu , Q. Fei , Y. Ai , P. C. He , H. Shi , X. Cui , R. Su , A. Klungland , G. Jia , J. Chen , C. He , Mol. Cell 2018, 71, 973. 30197295 10.1016/j.molcel.2018.08.011 PMC6151148

[80] E. Sendinc , D. Valle‐Garcia , A. Dhall , H. Chen , T. Henriques , J. Navarrete‐Perea , W. Q. Sheng , S. P. Gygi , K. Adelman , Y. Shi , Mol. Cell 2019, 75, 620. 31279659 10.1016/j.molcel.2019.05.030 PMC6688901

[81] E. Izaurralde , J. Stepinski , E. Darzynkiewicz , I. W. Mattaj , J. Cell Biol. 1992, 118, 1287. 1522107 10.1083/jcb.118.6.1287 PMC2289605

[82] C. J. Brown , I. McNae , P. M. Fischer , M. D. Walkinshaw , J. Mol. Biol. 2007, 372, 7. 17631896 10.1016/j.jmb.2007.06.033

[83] M. van Dülmen , N. Muthmann , A. Rentmeister , Angew. Chem. Int. Ed. 2021, 60, 13280. 10.1002/anie.202100352 PMC8250829 33751748

[84] K. A. Doxtader , Y. Nam , Mol. Cell 2019, 75, 417. 31398320 10.1016/j.molcel.2019.07.019 PMC6860360

[85] C. Schuberth‐Wagner , J. Ludwig , A. K. Bruder , A. M. Herzner , T. Zillinger , M. Goldeck , T. Schmidt , J. L. Schmid‐Burgk , R. Kerber , S. Wolter , J. P. Stümpel , A. Roth , E. Bartok , C. Drosten , C. Coch , V. Hornung , W. Barchet , B. M. Kümmerer , G. Hartmann , M. Schlee , Immunity 2015, 43, 41. 26187414 10.1016/j.immuni.2015.06.015 PMC7128463

[86] M. Müller‐McNicoll , K. M. Neugebauer , Nat. Rev. Genet. 2013, 14, 275. 23478349 10.1038/nrg3434

[87] V. Hornung , J. Ellegast , S. Kim , K. Brzózka , A. Jung , H. Kato , H. Poeck , S. Akira , K. K. Conzelmann , M. Schlee , S. Endres , G. Hartmann , Science 2006, 314, 994. 17038590 10.1126/science.1132505

[88] G. D. Williams , N. S. Gokhale , D. L. Snider , S. M. Horner , msSphere 2020, 5, e00202. 10.1128/mSphere.00202-20 PMC7227766 32404510

[89] Y. Furuichi , A. J. Shatkin , Adv. Virus Res. 2000, 55, 135. 11050942 10.1016/S0065-3527(00)55003-9 PMC7131690

[90] A. Pichlmair , O. Schulz , C. P. Tan , T. I. Näslund , P. Liljeström , F. Weber , C. Reis , Sousa , Science 2006, 314, 997. 17038589 10.1126/science.1132998

[91] Y. L. Wang , J. Ludwig , C. Schuberth , M. Goldeck , M. Schlee , H. T. Li , S. Juranek , G. Sheng , R. Micura , T. Tuschl , G. Hartmann , D. J. Patel , Nat. Struct. Mol. Biol. 2010, 17, 781. 20581823 10.1038/nsmb.1863 PMC3744876

[92] R. Züst , L. Cervantes‐Barragan , M. Habjan , R. Maier , B. W. Neuman , J. Ziebuhr , K. J. Szretter , S. C. Baker , W. Barchet , M. S. Diamond , S. G. Siddell , B. Ludewig , V. Thiel , Nat. Immunol. 2011, 12, 137. 21217758 10.1038/ni.1979 PMC3182538

[93] Y. M. Abbas , A. Pichmair , M. W. Górna , G. Superti‐Furga , B. Nagar , Nature 2013, 494, 60. 23334420 10.1038/nature11783 PMC4931921

[94] J. M. Holstein , D. Schulz , A. Rentmeister , Chem. Commun. 2014, 50, 4478. 10.1039/c4cc01549e 24664183

[95] J. M. Holstein , L. Anhäuser , A. Rentmeister , Angew. Chem. Int. Ed. 2016, 55, 10899. 10.1002/anie.201604107 27511141

[96] V. H. Cowling , Trends Biochem. Sci. 2019, 44, 183. 30679132 10.1016/j.tibs.2019.01.002 PMC6378647

[97] J. Jemielity , T. Fowler , J. Zuberek , J. Stepinski , M. Lewdorowicz , A. Niedzwiecka , R. Stolarski , E. Darzynkiewicz , R. E. Rhoads , RNA 2003, 9, 1108. 12923259 10.1261/rna.5430403 PMC1370475

[98] A. E. Pasquinelli , J. E. Dahlberg , E. Lund , RNA 1995, 1, 957. 8548660 PMC1369344

[99] J. Stepinski , C. Waddell , R. Stolarski , E. Darzynkiewicz , R. E. Rhoads , RNA 2001, 7, 1486. 11680853 PMC1370192

[100] E. Grudzien , M. Kalek , J. Jemielity , E. Darzynkiewicz , R. E. Rhoads , J. Biol. Chem. 2006, 281, 1857. 16257956 10.1074/jbc.M509121200

[101] M. Mockey , C. Gonçalves , F. P. Dupuy , F. M. Lemoine , C. Pichon , P. Midoux , Biochem. Biophys. Res. Commun. 2006, 340, 1062. 16403444 10.1016/j.bbrc.2005.12.105

[102] B. Miedziak , A. Dobieżyńska , Z. M. Darżynkiewicz , J. Bartkowska , J. Miszkiewicz , J. Kowalska , M. Warminski , M. Tyras , J. Trylska , J. Jemielity , E. Darzynkiewicz , R. Grzela , RNA 2020, 26, 58. 31658992 10.1261/rna.073304.119 PMC6913129

[103] A. R. Kore , M. Shanmugasundaram , I. Charles , A. V. Vlassov , T. J. Barta , J. Am. Chem. Soc. 2009, 131, 6364. 19385620 10.1021/ja901655p

[104] H. Y. Chen , D. L. Liu , A. Aditham , J. T. Guo , J. H. Huang , F. Kostas , K. Maher , M. J. Friedrich , R. J. Xavier , F. Zhang , X. Wang , Nat. Biotechnol. 2024, 43, 1128. 39313647 10.1038/s41587-024-02393-y PMC11929619

[105] A. Senthilvelan , T. Vonderfecht , M. Shanmugasundaram , I. Pal , J. Potter , A. R. Kore , Org. Lett. 2021, 23, 4133. 34008991 10.1021/acs.orglett.1c01037

[106] H. Ohno , S. Akamine , M. Mochizuki , K. Hayashi , S. Akichika , T. Suzuki , H. Saito , Nucleic Acids Res. 2023, 15, e34. 10.1093/nar/gkad019 PMC10085709 36731515

[107] A. Senthilvelan , T. Vonderfecht , M. Shanmugasundaram , J. Potter , A. R. Kore , Bioorg. Med. Chem. 2023, 77, 117128. 36516685 10.1016/j.bmc.2022.117128

[108] J. M. Holstein , D. Stummer , A. Rentmeister , Chem. Sci. 2015, 6, 1362. 29560223 10.1039/c4sc03182b PMC5811123

[109] N. Klöcker , L. Anhäuser , A. Rentmeister , ChemBioChem 2023, 24, e202200522. 36408753 10.1002/cbic.202200522 PMC10108117

[110] E. Darzynkiewicz , J. Stepinski , I. Ekiel , Y. Jin , D. Haber , T. Sijuwade , S. M. Tahara , Nucleic Acids Res. 1988, 16, 8953. 3174438 10.1093/nar/16.18.8953 PMC338645

[111] A. G. Seto , A. J. Zaug , S. G. Sobel , S. L. Wolin , T. R. Cech , Nature 1999, 402, 177. 10.1038/43694 10490028

[112] J. Mouaikel , C. Verheggen , E. Bertrand , J. Tazi , R. Bordonné , Mol. Cell 2002, 9, 891. 11983179 10.1016/s1097-2765(02)00484-7

[113] T. Monecke , A. Dickmanns , R. Ficner , Nucleic Acids Res. 2009, 37, 3865. 19386620 10.1093/nar/gkp249 PMC2709555

[114] I. Kocmik , K. Piecyk , M. Rudzinska , A. Niedzwiecka , E. Darzynkiewicz , R. Grzela , M. Jankowska‐Anyszka , Cell Cycle 2018, 17, 1624. 29954234 10.1080/15384101.2018.1486164 PMC6133335

[115] R. Grzela , K. Piecyk , A. Stankiewicz‐Drogon , P. Pietrow , M. Lukaszewicz , K. Kurpiejewski , E. Darzynkiewicz , M. Jankowska‐Anyszka , RNA 2023, 29, 200. 36418172 10.1261/rna.079460.122 PMC9891257

[116] N. V. Cornelissen , R. Mineikaitė , M. Ergüven , N. Muthmann , A. Peters , A. Bartels , A. Rentmeister , Chem. Sci. 2023, 14, 10962. 37829022 10.1039/d3sc03822j PMC10566477

[117] E. Darzynkiewicz , J. Stepinski , I. Ekiel , C. Goyer , N. Sonenberg , A. Temeriusz , Y. Jin , T. Sijuwade , D. Haber , S. M. Tahara , Biochemistry 1989, 28, 4771. 2548592 10.1021/bi00437a038

[118] R. Wojcik , M. R. Baranowski , L. Markiewicz , D. Kubacka , M. Bednarczyk , N. Baran , A. Wojtczak , P. J. Sikorski , J. Zuberek , J. Kowalska , J. Jemielity , Pharmaceutics 2021, 13, 1941. 34834356 10.3390/pharmaceutics13111941 PMC8623273

[119] M. Warminski , E. Trepkowska , M. Smietanski , P. J. Sikorski , M. R. Baranowski , M. Bednarczyk , H. Kedzierska , B. Majewski , A. Mamot , D. Papiernik , A. Popielec , R. A. Serwa , B. A. Shimanski , P. Sklepkiewicz , M. Sklucka , O. Sokolowska , T. Spiewla , D. Toczydlowska‐Socha , Z. Warminska , K. Wolosewicz , J. Zuberek , J. S. Mugridge , D. Nowis , J. Golab , J. Jemielity , J. Kowalska , J. Am. Chem. Soc. 2024, 146, 8149. 38442005 10.1021/jacs.3c12629 PMC10979456

[120] J. Jemielity , J. Kowalska , A. M. Rydzik , E. Darzynkiewicz , New J. Chem. 2010, 34, 829.

[121] M. Warminski , P. J. Sikorski , J. Kowalska , J. Jemielity , in Phosphate Labeling and Sensing in Chemical Biology, Springer International Publishing AG, London, UK 2017, pp. 211–239.

[122] A. Kuhn , M. Diken , S. Kreiter , A. Selmi , J. Kowalska , J. Jemielity , E. Darzynkiewicz , C. Huber , O. Tureci , U. Sahin , Gene Ther. 2010, 17, 961. 20410931 10.1038/gt.2010.52

[123] E. Grudzien‐Nogalska , J. Jemielity , J. Kowalska , E. Darzynkiewicz , R. E. Rhoads , RNA 2007, 13, 1745. 17720878 10.1261/rna.701307 PMC1986804

[124] J. Kowalska , M. Lukaszewicz , J. Zuberek , E. Darzynkiewicz , J. Jemielity , ChemBioChem 2009, 10, 2469. 19739194 10.1002/cbic.200900522

[125] J. Kowalska , A. W. del Nogal , Z. M. Darzynkiewicz , J. Buck , C. Nicola , A. N. Kuhn , M. Lukaszewicz , J. Zuberek , M. Strenkowska , M. Ziemniak , M. Maciejczyk , E. Bojarska , R. E. Rhoads , E. Darzynkiewicz , U. Sahin , J. Jemielity , Nucleic Acids Res. 2014, 42, 10245. 25150148 10.1093/nar/gku757 PMC4176373

[126] W. Su , S. Slepenkov , E. Grudzien‐Nogalska , J. Kowalska , M. Kulis , J. Zuberek , M. Lukaszewicz , E. Darzynkiewicz , J. Jemielity , R. E. Rhoads , RNA 2011, 17, 978. 21447710 10.1261/rna.2430711 PMC3078746

[127] M. Strenkowska , R. Grzela , M. Majewski , K. Wnek , J. Kowalska , M. Lukaszewicz , J. Zuberek , E. Darzynkiewicz , A. N. Kuhn , U. Sahin , J. Jemielity , Nucleic Acids Res. 2016, 44, 9578. 27903882 10.1093/nar/gkw896 PMC5175369

[128] B. A. Wojtczak , P. J. Sikorski , K. Fac‐Dabrowska , A. Nowicka , M. Warminski , D. Kubacka , E. Nowak , M. Nowotny , J. Kowalska , J. Jemielity , J. Am. Chem. Soc. 2018, 140, 5987. 29676910 10.1021/jacs.8b02597

[129] A. M. Rydzik , M. Warminski , P. J. Sikorski , M. R. Baranowski , S. Walczak , J. Kowalska , J. Zuberek , M. Lukaszewicz , E. Nowak , T. D. W. Claridge , E. Darzynkiewicz , M. Nowotny , J. Jemielity , Nucleic Acids Res. 2017, 45, 8661. 28666355 10.1093/nar/gkx569 PMC5587727

[130] A. M. Rydzik , M. Kulis , M. Lukaszewicz , J. Kowalska , J. Zuberek , Z. M. Darzynkiewicz , E. Darzynkiewicz , J. Jemielity , Bioorg. Med. Chem. 2012, 20, 1699. 22316555 10.1016/j.bmc.2012.01.013

[131] S. Walczak , P. J. Sikorski , R. Kasprzyk , J. Kowalska , J. Jemielity , Org. Biomol. Chem. 2018, 16, 6741. 30187040 10.1039/c8ob01720d

[132] M. Kozarski , K. Drazkowska , M. Bednarczyk , M. Warminski , J. Jemielity , J. Kowalska , RSC Adv. 2023, 13, 12809. 37114020 10.1039/d3ra00026e PMC10126820

[133] M. L. Winz , A. Samanta , D. Benzinger , A. Jäschke , Nucleic Acids Res. 2012, 40, e78. 22344697 10.1093/nar/gks062 PMC3378897

[134] C. Y. Jao , A. Salic , Proc. Natl. Acad. Sci. U.S.A. 2008, 105, 15779. 18840688 10.1073/pnas.0808480105 PMC2572917

[135] J. Schoch , S. Ameta , A. Jäschke , Chem. Comm. 2011, 47, 12536. 22002170 10.1039/c1cc15476a

[136] A. Mamot , P. J. Sikorski , M. Warminski , J. Kowalska , J. Jemielity , Angew. Chem. Int. Ed. 2017, 56, 15628. 10.1002/anie.201709052 29048718

[137] D. Schulz , J. M. Holstein , A. Rentmeister , Angew. Chem. Int. Ed. 2013, 52, 7874. 10.1002/anie.201302874 23794451

[138] M. Inagaki , N. Abe , Z. M. Li , Y. Nakashima , S. Acharyya , K. Ogawa , D. Kawaguchi , H. Hiraoka , A. Banno , Z. Y. Meng , M. Tada , T. Ishida , P. Lyu , K. Kokubo , H. Murase , F. Hashiya , Y. Kimura , S. Uchida , H. Abe , Nat. Commun. 2023, 14, 17. 37169757 10.1038/s41467-023-38244-8 PMC10175277

[139] Z. Meng , Y. Nakashima , M. Inagaki , Z. Li , S. Acharyya , F. Hashiya , N. Abe , Y. Kimura , H. Abe , Bull. Chem. Soc. Jpn. 2025, 98.

[140] S. Ogasawara , ChemBioChem 2014, 15, 2652. 25351829 10.1002/cbic.201402495

[141] S. Ogasawara , ACS Chem. Biol. 2017, 12, 351. 28049292 10.1021/acschembio.6b00684

[142] N. Klöcker , F. P. Weissenboeck , M. van Dülmen , P. Špaček , S. Hüwel , A. Rentmeister , Nat. Chem. 2022, 14, 905. 35725774 10.1038/s41557-022-00972-7 PMC7613264

[143] H. Schepers , G. Dahm , M. Sumser , S. Hüwel , A. Rentmeister , Chem. Sci. 2025. 10.1039/d5sc01999k PMC12230757 40630626

[144] F. P. Weissenboeck , M. Pieper , H. Schepers , S. Hötte , N. Klöcker , S. Hüwel , A. van Impel , S. Schulte‐Merker , A. Rentmeister , Commun. Chem. 2025. 10.1038/s42004-025-01411-7 PMC11743775 39828804

[145] A. Bollu , N. Klöcker , P. Špaček , F. P. Weissenboeck , S. Hüwel , A. Rentmeister , Angew. Chem. Int. Ed. 2023, 62, e202209975. 10.1002/anie.202209975 PMC10107135 36417319

[146] A. Bollu , H. Schepers , N. Klöcker , M. Erguven , A. M. Lawrence‐Dörner , A. Rentmeister , Chem. Eur. J. 2024, 30, e202303174. 37883670 10.1002/chem.202303174

[147] F. P. Weissenboeck , N. Klöcker , P. Spacek , S. Hüwel , A. Rentmeister , ACS Omega 2024, 9, 12810. 38524462 10.1021/acsomega.3c08505 PMC10955689

[148] C. Kühling , W. Teich , B. Terzi , H. Schepers , S. Hüwel , A. Rentmeister , ChemistryEurope 2025, 3, e202500178.

[149] M. Warminski , K. Grab , K. Szczepanski , T. Spiewla , J. Zuberek , J. Kowalska , J. Jemielity , Adv. Sci. 2024, 11, 2400994. 10.1002/advs.202400994 PMC11423160 39049186

